# Diagnostic prediction models for spinal fractures in individuals with spinal pain or trauma: a systematic review and meta-analysis

**DOI:** 10.1016/j.eclinm.2025.103456

**Published:** 2025-08-26

**Authors:** Daniel Feller, Roel Wingbermühle, Edwin H.G. Oei, Bart W. Koes, Alessandro Chiarotto

**Affiliations:** a Department of General Practice, Erasmus MC, University Medical Centre, Rotterdam, the Netherlands; b Provincial Agency for Health of the Autonomous Province of Trento, Trento, Italy; c Department of Physiotherapy and Rehabilitation Sciences, SOMT University of Physiotherapy, Amersfoort, the Netherlands; d Department of Radiology & Nuclear Medicine, Erasmus MC, University Medical Centre, Rotterdam, the Netherlands; e Research Unit of General Practice, Department of Public Health and the Centre for Muscle and Joint Health, University of Southern Denmark, Odense, Denmark

**Keywords:** Spinal fracture diagnosis, Diagnostic prediction models, Clinical decision rules, Canadian C-spine Rule

## Abstract

**Background:**

Multivariable diagnostic models are often used to identify spinal fractures in patients with spinal pain and/or trauma. However, their performance and clinical utility remain uncertain. We aimed to evaluate the performance of diagnostic models for detecting spinal fractures in individuals with spinal pain and/or trauma.

**Methods:**

In this systematic review and meta-analysis, we searched MEDLINE, EMBASE, and Web of Science on April 15, 2024 and May 27, 2024 for relevant work published since database inception. The first search included only studies on spinal pain and the second additionally included spinal trauma studies, following a protocol adjustment during screening. A search update was performed on May 19, 2025. An expert librarian assisted in developing the search strategy, which was limited to work published in English, Italian, and Dutch. We also performed backward and forward citation tracking. We included studies that developed and/or externally validated multivariable diagnostic prediction models for spinal fractures. Two independent reviewers screened studies for eligibility, extracted data using the CHARMS checklist, and assessed the risk of bias using the PROBAST. The certainty of evidence was evaluated using the GRADE approach. The protocol was registered in PROSPERO, CRD42024539898.

**Findings:**

We included 27 studies encompassing 34 diagnostic models. All models showed an overall high risk of bias, while the concerns about their applicability varied due to the frequent use of spinal injuries as the outcome instead of explicitly addressing spinal fractures. Meta-analyses of ten studies that externally validated the Canadian C-spine Rule in adults presenting with trauma to emergency departments or trauma centres demonstrated, with very low certainty of the evidence, excellent sensitivity (0.999; 95% CI 0.976–1), an high area under the curve (0.850; 95% CI 0.720–0.970), and a low specificity (0.188; 95% CI 0.063–0.443). We estimated a pooled non-statistically significant positive likelihood ratio of 1.230 (95% CI 0.978–1.548) and a negative likelihood ratio of 0.007 (95% CI 0.001–0.082) for the same model. Other models for traumatic cervical fractures and osteoporotic fractures showed promise but lacked external validation or sufficient reporting on calibration and discrimination measures (with low to very low certainty of the evidence). No models for thoracolumbar fractures were deemed ready to be used clinically.

**Interpretation:**

Although the Canadian C-spine Rule shows potential for screening traumatic cervical fractures, the very low to low certainty of the evidence limits confidence in its accuracy and appropriateness for clinical use. We did not identify any externally validated models suitable for clinical use regarding osteoporotic or traumatic fractures of the thoracolumbar spine, and traumatic fractures of the cervical spine in non-emergency settings. Future research with rigorous methodological and statistical approaches should aim to fill these knowledge gaps.

**Funding:**

None.

**Research in context**

**Evidence before this study**

Accurate screening for spinal fractures in individuals presenting with spinal pain or trauma is essential to ensure the timely identification of true cases while avoiding unnecessary imaging. Diagnostic prediction models, which combine the joint effects of multiple clinical variables using statistical methods or machine learning algorithms to estimate fracture probability, are increasingly recommended for this purpose. In February 2024, we searched MEDLINE (via PubMed) from database inception for systematic reviews of diagnostic prediction models for spinal fractures, using a combination of relevant terms. The search yielded 813 results, but none included a systematic review specifically focused on diagnostic prediction models. Therefore, to the best of our knowledge, no prior systematic reviews have synthesised the performance of diagnostic prediction models for diagnosing any type of spinal fracture.

**Added value of this study**

We conducted a systematic review of the literature, identifying 27 studies reporting on 34 developed and/or externally validated diagnostic models. A meta-analysis of the Canadian C-spine Rule for screening traumatic cervical fractures in emergency departments (10 studies; 35,544 patients; 1213 events) yielded a pooled sensitivity of 0.999 (95% CI: 0.976–1) and a specificity of 0.188 (95% CI: 0.063–0.443), with substantial heterogeneity. According to the GRADE approach, the certainty of the evidence was rated as very low. No validated models suitable for clinical use were identified for osteoporotic fractures, traumatic thoracolumbar fractures, or traumatic cervical fractures in non-emergency settings.

**Implications of all the available evidence**

The Canadian C-spine Rule may assist in ruling out cervical fractures in emergency settings due to its high sensitivity; however, its low specificity and the very low to low certainty of the supporting evidence limit its overall reliability. In non-emergency settings, such as primary care or outpatient clinics, a substantial evidence gap exists, with no validated diagnostic models currently available for spinal fractures. Furthermore, even within emergency care, no validated models have been developed for traumatic thoracolumbar or osteoporotic fractures. This underscores a pressing need to develop and validate diagnostic models for traumatic thoracolumbar fractures, osteoporotic fractures, and cervical fractures in non-emergency contexts.

## Introduction

Spinal disorders are one of the leading causes of disability worldwide, significantly affecting individuals' quality of life and placing a burden on healthcare systems and societies.^1^ A large majority of spinal conditions are classified as non-specific, meaning they do not have a certain disease or structural underlying cause.^2^ Nevertheless, a minority of patients present with serious spinal pathologies that require immediate attention.^2^^,^^3^ Among these, spinal fractures are the most common serious pathology, with an estimated incidence in the United States of 10.14 cases per 100,000 person-years.^3^^,^^4^

Spinal fractures are typically classified into three main types: traumatic fractures, osteoporotic (compression) fractures, and stress fractures, though the latter are far less common than the other two. Although all spinal fractures represent serious conditions, they occur in markedly different clinical contexts and have distinct consequences. Traumatic fractures typically result from high-energy injuries and often require urgent evaluation and intervention. In contrast, osteoporotic fractures occur in patients with reduced bone density, frequently after minimal or no trauma, and can sometimes be managed conservatively. The urgency of accurate diagnosis also varies between these types, with traumatic fractures often needing immediate imaging and stabilisation, while osteoporotic fractures may follow a different diagnostic and therapeutic pathway.^5^^,^^6^ According to the American College of Radiology (ACR) Appropriateness Criteria, various imaging techniques are recommended as the reference standard to diagnose these fractures.^5^, ^6^, ^7^, ^8^ Radiographs are advised for identifying osteoporotic fractures, computed tomography (CT) for traumatic fractures, and either CT or magnetic resonance imaging (MRI) for stress fractures.^5^, ^6^, ^7^, ^8^, ^9^ However, these imaging procedures can be expensive and carry various risks for patients, including radiation exposure, overdiagnosis, and potential delays in care. Given these challenges, it is crucial to screen people who present with spinal pain or trauma for fractures, with the aim of identifying all patients with fractures while minimising unnecessary imaging.^3^ Unfortunately, individual components of a patient's history and physical examination often lack the diagnostic validity needed for a reliable screening of spinal fractures.^10^^,^^11^ Due to this reason, diagnostic prediction models that combine the joint effects of multiple variables using statistical methods or machine learning algorithms to estimate the likelihood of fractures are increasingly recommended.^12^ A well-known example is the Canadian C-spine Rule, a clinical decision-making tool designed to identify high-risk people who need imaging for cervical spine injuries.^13^

Although prediction models exhibit great promise, numerous systematic reviews in the spinal pain field have shown that many of these models do not yet meet the necessary minimum criteria for clinical implementation.^14^, ^15^, ^16^ For example, statistical measures of discrimination, calibration, and clinical utility are crucial for accurately assessing a model's performance.^17^ However, many studies often overlook one or more of these important metrics, resulting in gaps in evaluating the model's true performance.^18^

To the best of our knowledge, there are no existing systematic reviews synthesising the performance of diagnostic prediction models for diagnosing any type of spinal fracture. Considering that various spinal fracture types exhibit unique clinical symptoms, like acute trauma for high-energy fractures and trauma-free pain in osteoporotic cases, we found it essential to regard both trauma and spinal pain as pertinent presentations. Therefore, this systematic review aims to summarise the diagnostic accuracy of multivariable diagnostic models for suspected spinal fractures in individuals with spinal pain and/or trauma, presenting the data separately based on spinal region and fracture type to account for heterogeneity in clinical presentation and diagnostic approaches.

## Methods

### Study design and ethics

The protocol for this systematic review and meta-analysis was registered in PROSPERO (CRD42024539898). We used the “Transparent reporting of multivariable prediction models for individual prognosis or diagnosis–Systematic Reviews and Meta-Analysis” (TRIPOD-SRMA) checklist to report the present manuscript.^19^

For this study, we did not seek ethical approval because it is a systematic review of previously published literature and does not involve the collection or analysis of individual patient data. Accordingly, the requirement for written informed consent was waived.

### Data sources and searches

Literature searches were conducted on April 15 and May 27, 2024, in MEDLINE (via Ovid), EMBASE, and Web of Science from inception. The first search targeted studies on spinal pain, while the second included spinal trauma studies, following a protocol adjustment during screening. Backward and forward citation tracking was performed using the Web of Science. The search was updated on May 19, 2025. An expert librarian assisted in developing the search strategy, which was limited to English, Italian, and Dutch articles (full details in Supplementary Material 1). Two independent reviewers (D.F. and R.W.) screened studies, resolving disagreements by consensus or with a third reviewer (A.C.). The selection process was managed using Rayyan QCRI.^20^

### Study selection

We included observational studies (cohort and case–control) and randomised controlled trials that developed and/or validated multivariable diagnostic models for identifying spinal fractures in people with spinal pain or trauma, regardless of the imaging modality used for diagnosis. We defined diagnostic models as equations or algorithms that combine multiple variables to assess their combined effect on diagnosing spinal fractures.^21^ Consequently, we excluded any equations or algorithms that grouped the variables in the final model without employing statistical methodologies, like, for example, the “National Emergency X-Radiography Utilization Study” (NEXUS) criteria.^22^ Studies involving mixed populations, like those assessing spine injuries generally rather than specifically fractures, were included only if over 80% met our criteria. Alternatively, studies with separate analyses for the specific data of interest were also included.

### Data extraction and quality assessment

After a piloting phase on five studies, two reviewers independently (D.F. and R.W., or D.F. and A.C.) extracted data using items adapted from the “Critical Appraisal and Data Extraction for Systematic Reviews of Prediction Modelling Studies” (CHARMS) checklist.^23^ To assess a model's performance, we extracted measures of discrimination (e.g., c-statistic), calibration (e.g., calibration slope), classification (e.g., sensitivity and specificity), clinical utility (e.g., decision curve analysis), and overall performance (e.g., R-squared).

The risk of bias and applicability of the included studies was assessed independently by two reviewers (D.F. and R.W., or D.F. and A.C.) using the “Prediction model Risk Of Bias Assessment Tool” (PROBAST).^24^ The risk of bias in the outcome domain was rated high when the imaging used for diagnosing a traumatic fracture was not a CT scan.^24^ Additionally, the applicability of the outcome domain was considered as a high concern if the model was designed for a broader range of spinal injuries (such as dislocations and ligamentous instabilities) rather than specifically for diagnosing fractures.

### Data synthesis and statistical analysis

We used Meta-Disc 2.0 to pool the classification measures (i.e., sensitivity and specificity) of the Canadian C-spine Rule in a bivariate random effects meta-analysis.^25^^,^^26^ To address zero or near-zero cells in sensitivity or specificity, we applied a continuity correction of 0.5 to ensure model convergence and stability. To pool the discrimination measures of the same model, we used the metafor package in R (version 4.4.3)^27^ to conduct a univariate random-effects model, with the restricted maximum likelihood method used to estimate the between-study variance. To assess the heterogeneity in the meta-analyses, a prediction interval was calculated.^28^^,^^29^ When a study reported multiple external validation results for the Canadian C-spine Rule due to applications by different healthcare professionals, we included in the meta-analysis only the data from applications performed by medical personnel. We conducted a subgroup analysis to compare the results between studies that specifically evaluated fractures as the primary outcome, and those that assessed cervical spine injuries in general; a sensitivity analysis was also performed by excluding studies that included a mixed population of children and adults. Both the subgroup and the sensitivity analysis were not pre-planned in the protocol. We did not assess the risk of small-study effects (publication bias) because the limited number of studies included in the meta-analyses would have made the interpretation of funnel plots unreliable. The other models were not included in a meta-analysis due to the absence of multiple studies evaluating the same model, which precluded meaningful data pooling. Thresholds of acceptable or clinically useful levels of predictive ability (e.g., sensitivity, specificity, discrimination, calibration) were not pre-specified.

We assessed the certainty of the evidence for the meta-analysis results conducted using an adapted version of the “Grading of Recommendations Assessment, Development and Evaluation” (GRADE) approach for prognostic factors.^30^ Supplementary Material 2 reports how we applied the GRADE criteria.

### Role of the funding source

This study did not receive any specific funding.

## Results

### Characteristics of the included studies

**Fig. 1:**
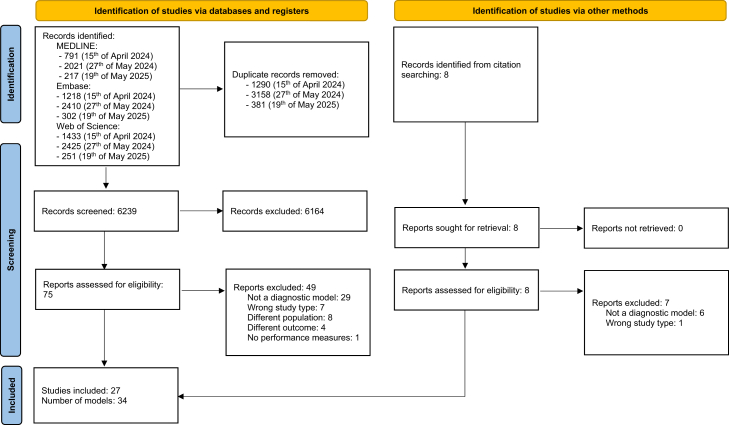
**PRISMA flowchart**.

Fig. 1 shows the study selection process. After screening the title/abstract and full text, we included 27 studies that covered 34 developed and/or externally validated models. The updated search, performed on May 19, 2025, yielded no additional studies that were eligible for inclusion.

Table 1 summarises the characteristics of the 27 included studies. Among them, 10 (37%) developed diagnostic models, 5 (19%) combined development with internal validation, 3 (11%) developed a model while externally validating another, and 9 (33%) exclusively validated the Canadian C-spine Rule. Regarding the performance measures, 17 models (50%) were assessed by classification alone, 9 (26%) by discrimination and classification, 3 (9%) by discrimination only, 3 (9%) by calibration, discrimination, and classification, and 1 (3%) by overall performance, discrimination, and classification. No studies reported clinical utility measures. Two studies included only females; in the rest, the median percentage of males was 57% (range 34–64). The median sample size was 1296 (range 80–8924), with a median of 64 outcomes (range 7–540). The median fracture incidence was 6.1%. Supplementary Materials 3–9 report the complete characteristics of the included studies. Supplementary Material 10 reports the certainty in the evidence assessed with the GRADE approach for models other than the Canadian C-spine Rule.

**Table 1:** Main study characteristics.

First author (year); model information	Setting	Number of models developed/validated	Cohort type	Spine location	Outcome	Sample size/number of events	Age	Sex
Athinartrattanapong (2021); development of a new model	Emergency department	1 D	Cross-sectional study	Cervical spine	Traumatic cervical spinal injury	375 (29 cervical spine injuries and 346 non-cervical spine injuries)	Age ≥65 years: Cases: 11 Non-cases: 124	Cases: 13 males (44.8%) Non-cases: 212 males (61.3%)
Bandiera (2003); external validation of the Canadian C-spine	Emergency department	1 V	Cross-sectional study	Cervical spine	Traumatic cervical spinal injury	6265/64 cervical spine injury	Mean 36.6 (SD 16) years	3.177 males (50.6%)
Bub (2005); development and internal validation of a new model	Trauma center	1 D + IV	Case—control study	Cervical spine	Traumatic fracture	210 (103 fractures and 107 non-fractures)	Cases: mean 77 (range 65–102) years Controls: mean 76 (range 65–101) years	117 males (55.7%)
Caltili (2017); external validation of the Canadian C-spine	Emergency department	1 V	Case—control study	Cervical spine	Traumatic fracture	2442 patients (338 fractures and 2104 non-fractures)	- 0–9 years: 162 (7%) - 10–19 years: 283 (12%) - 20–29 years: 577 (24%) - 30–39 years: 443 (18%) - 40–49 years: 333 (14%) - 50–64 years: 226 (9%) - ≥ 65 years: 418 (17%)	1566 males (64%)
Clark (2016); development of a new model	Primary (around 80% of the included patients) and secondary care	1 D	Case—control study	Thoracic spine	Osteoporotic fracture	197 (64 fractures and 133 non-fractures)	Cases: mean 76.9 (IQR 71.2–83.5) years Controls: mean 71.7 (IQR 67.0–78.0) years	All females by inclusion criteria
Coffrey (2015); external validation of the Canadian C-spine	Emergency department	1 V	Cross-sectional study	Cervical spine	Traumatic cervical spine injury	1420/8 cervical spine injuries	Not reported	Not reported
Cook (2013); development of a new model	Tertiary care (Department of surgery)	1 D	Retrospective cohort study	Cervical spine	Fracture (all types)	162/11 fractures	Age ≤33: 9 patients (5%)	79 males (49%)
Duane (2011); development of a new model; external validation of the Canadian C-spine	Trauma center	1 D 1 V	Cross-sectional study	Cervical spine	Traumatic fracture	3201/192 fractures	Fracture group: mean 42.7 (SD 19.0) years Non-fracture group: mean 37.8 (SD 17.5) years	2051 males (64.1%)
Duane (2013); development of a new model; external validation of the Canadian C-spine	Trauma center	1 D 1 V	Cross-sectional study	Cervical spine	Traumatic fracture	5182/324 fractures	Fracture group: mean 43.9 (SD 18.8) years Non-fracture group: mean 38.4 (SD 17.5) years	3293 males (63.5%)
Ehrlich (2009); external validation of the Canadian C-spine	Pediatric trauma center	1 V	Retrospective cohort study	Cervical spine	Traumatic cervical spine injury	125/7 cervical spine injuries	Mean 4.3 (SD 3.1)	72 males (57%)
Engelbart (2021); development and internal validation of two new model	Trauma center	2 D + IV	Cross-sectional study	Cervical spine	Traumatic cervical spine injury	Training set: 707/75 cervical spine injuries Validation set: 1605/178 cervical spine injuries	Training set: mean 81.3 (SD 8.4) years Validation set: mean 80.9 (SD 8.4) years	Training set: 273 (38.6%) males Validation set: 631 (39.3%) males
Enthoven (2016); development of a new model	General practices	1 D	Prospective cohort study	Thoracic and lumbar spine	Fracture (all types)	669/33 fractures	Mean 66 (SD 7.7) years	269 males (40%)
Ghelichkhani (2021); external validation of the Canadian C-spine	Emergency department	1 V	Cross-sectional study	Cervical spine	Traumatic cervical spine injury	673/61 cervical spine injuries	Mean 34.3 (SD 19.4) years	466 males (69.2%)
Henschke (2009); development of a new model	Primary care	1 D	Prospective cohort study	Lumbar spine	Fracture (all types)	1172/8 fractures	Mean 43.97 (SD 15.1) years	626 males (53.4%)
Hercz (2019); development of a new model	Emergency department	1 D	Retrospective cohort study	Thoracolumbar spine	Traumatic thoracolumbar spine injury	1049/36 thoracolumbar spine injuries	Median 46 (range 33–57) years	561 males 53.4%
Ikemoto (2022); development of a new model	Secondary care	1 D	Cross—sectional study	Lower thoracic and lumbar spine	Osteoporotic fracture	80/40 fractures	Patients with a fracture: mean 79.5 (SD 8.3) years Patients without a fracture: mean 76.7 (SD 7.6) years	27 males (34%)
Inaba (2015); development of two new models	Trauma centers	2 D	Cross-sectional study	Thoracolumbar spine	Traumatic thoracolumbar spine injury	3065/264 thoracolumbar spine injuries	Mean 43.5 (SD 19.8) years	2031 (66.3%) males
Inagaki (2018); development of a new model; external validation of the same model	Emergency department	1 D 1 V	Cross-sectional study	Cervical spine	Traumatic cervical spine injury	927/38 cervical spine injuries	Median 59 (IQR 36–75) years	587 (63.3%) males
Khera (2022); development and internal validation of a new model	Primary care	1 D + IV	Cross—sectional study	Thoracic and lumbar spine	Osteoporotic fracture	1601/202 fractures	Mean 73.9 (range 65.4–96.8) years	All females
Leonard (2011); development and internal validation of two new models	Pediatric Emergency Department	2 D + IV	Case—control study	Cervical spine	Traumatic cervical spine injury	Cases: 540 cervical spine injuries Random controls: 1060 Mechanism of injury controls: 1012 Emergency medical services controls: 702	Cases: - 0–2: 27 - 2–8: 140 - 8–16: 373 Random controls: - 0–2: 116 - 2–8: 318 - 8–16: 626 Mechanism of injury controls: - 0–2: 41 - 2–8: 264 - 8–16: 707 Emergency medical services controls: - 0–2: 34 - 2–8: 173 - 8–16: 495	Cases: 344 males (64%) Random controls: 634 males (60%) Mechanism of injury controls: 620 males (61%) Emergency medical services controls: 414 males (59%)
Roux (2007); development of a new model	Not clear	1 D	Cross—sectional study	Thoracic and lumbar spine (from T4 to L5)	Osteoporotic fracture	397/not clear the number of patients with a fracture	Mean 74.3 (SD 5.5) years	All females
Singh (2011); development of a new model	Emergency Department	1 D	Case – control study	Thoracic spine	Traumatic fracture	773 (261 fractures and 512 non-fratures)	Mean 41 years	540 males (70%)
Stiell (2001); development and internal validation of two new models	Emergency Department	2 D + IV	Cross—sectional study	Cervical spine	Traumatic cervical spine injury	8924/515 cervical spine injuries	Mean 36.7 (SD 16) years	4600 males (51.5%)
Stiell (2003); external validation of the Canadian C-spine	Emergency Department	1 V	Cross-sectional study	Cervical spine	Traumatic cervical spine injury	8283/169 cervical spine injuries	Mean 37.6 (SD 16) years	4328 males (52.3%)
Stiell (2010); external validation of the Canadian C-spine	Emergency Departments	1 V	Cross-sectional study	Cervical spine	Traumatic cervical spine injury	3411/41 cervical spine injuries	Mean 41 (SD 18) years	1687 males (36.4%)
Vaillancourt (2009); external validation of the Canadian C-spine	Out-of-hospital	1 V	Cross-sectional study	Cervical spine	Traumatic cervical spine injury	1947/12 cervical spine injuries for paramedics 1629/12 cervical spine injuries for study investigators	Median 39 years (IQR 26–52, range 16–103) years	1403 males (49.3%)
Vaillancourt (2023); external validation of the Canadian C-spine	Out-of-hospital	1 V	Cross-sectional study	Cervical spine	Traumatic cervical spine injury	4021/11 cervical spine injuries for paramedics 3842/11 cervical spine injuries for study investigators	Mean 42.9 years (range 16–99) years	1881 males (46.9%)

D, Development, IV, Internal validation, V, External validation.

### Risk of bias and applicability concerns of the included models

**Fig. 2:**
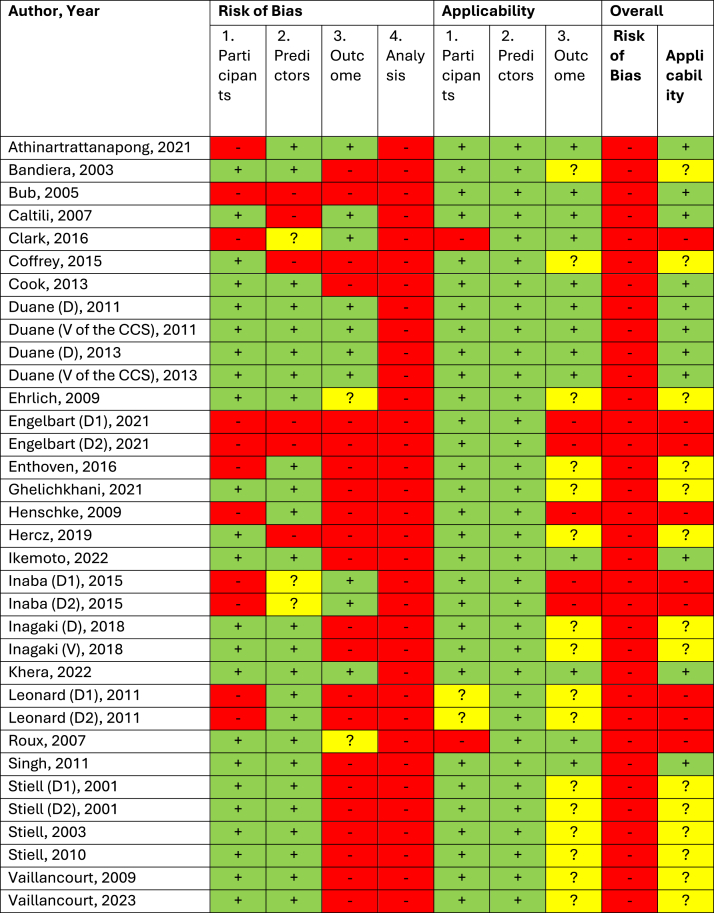
**Results of the risk of bias evaluation**. D, Developed model; V, Validated model; D1, First model developed; D2, Second model developed; CSS, Canadian C-Spine Rule.

All models had a high overall risk of bias (Fig. 2), with every model showing high bias in the analysis domain. In line with the PROBAST signalling questions for this domain, this assessment was primarily due to several common methodological limitations: the use of inappropriate methods for handling missing data (e.g., complete case analysis without justification), absence of internal validation (e.g., no cross-validation or bootstrapping), inadequate reporting or omission of key performance measures (particularly calibration metrics), and a high risk of overfitting due to small sample sizes relative to the number of candidate predictors. Additionally, 22 models (65%) had a high bias in the outcome domain due to suboptimal fracture diagnosis methods, such as using radiographs for traumatic fractures. Regarding applicability, 11 models (32%) had low concerns, 14 (41%) were unclear, and 9 (27%) had high concerns, primarily due to outcome definitions that included broader cervical spine injuries beyond fractures.

### Canadian C-spine rule

Stiell et al. (2001)^13^ developed the Canadian C-spine Rule, demonstrating a sensitivity of 100% (95% CI 98%–100%) and a specificity of 42.5% (95% CI 40%–44%) in identifying cervical spinal injuries in adults after trauma.

#### External validation in adults in the emergency department setting

**Fig. 3:**
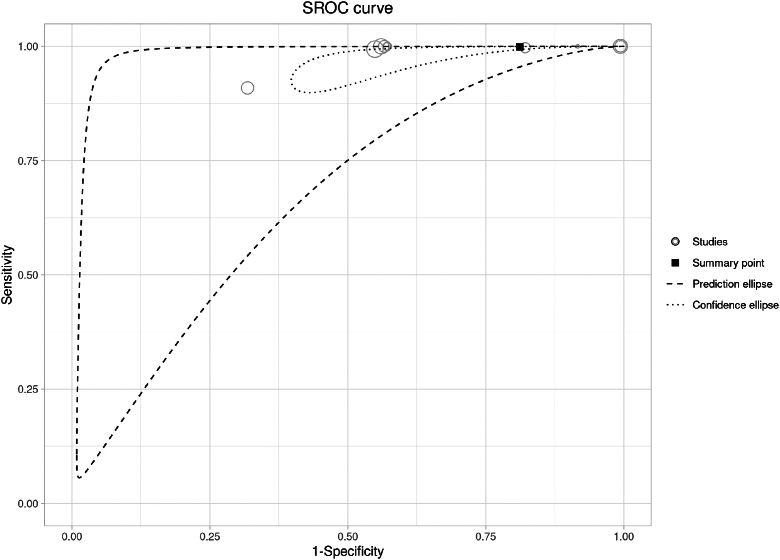
**Summary Receiver Operating Characteristic (SROC) curve of the meta-analysis for the external validation studies of the Canadian C-spine Rule**. Each circle represents an individual study included in the meta-analysis. The black square indicates the summary point (pooled sensitivity and specificity). The dashed ellipse represents the 95% prediction region, reflecting the expected range of sensitivity and specificity in future studies. The dotted ellipse indicates the 95% confidence region around the summary point. NA, not applicable.

Ten studies^31^, ^32^, ^33^, ^34^, ^35^, ^36^, ^37^, ^38^, ^39^ externally validated the Canadian C-spine Rule in adults with cervical injuries admitted to emergency departments or trauma Centres. We performed a bivariate meta-analysis of the Canadian C-spine Rule's classification measures (i.e., sensitivity and specificity) at external validation for adults. The main analysis, which included 10 studies and 35,544 patients (1213 events), showed a pooled sensitivity of 0.999 (95% CI: 0.976–1) and a specificity of 0.188 (95% CI: 0.063–0.443). The positive likelihood ratio was 1.230 (95% CI: 0.978–1.548), the negative likelihood ratio was 0.007 (95% CI: 0.001–0.082), and the false positive rate was 0.812 (95% CI: 0.557–0.937). Fig. 3 illustrates the prediction ellipse for the estimated pooled sensitivity and specificity. Table 2 includes a subgroup analysis comparing studies that focused exclusively on fractures evaluated through CT scans versus those that considered a broader outcome (i.e., cervical spine injury). In the subgroup of studies assessing fractures through CT (3 studies, 10,825 patients), the sensitivity was 1.00 (95% CI: 0.996–1), and the specificity was 0.020 (95% CI: 0.005–0.072). In contrast, studies using cervical spine injury as the outcome (7 studies, 24,719 patients) showed a sensitivity of 0.998 (95% CI: 0.924–1) and a specificity of 0.398 (95% CI: 0.216–0.613). Table 2 also reports the findings of a sensitivity analysis that excluded one study involving a mixed population of children and adults. When this study was excluded (9 studies, 33,102 patients), the pooled sensitivity was 1.00 (95% CI: 0.95–1), while specificity was 0.189 (95% CI: 0.056–0.479). Table 3 presents the certainty of evidence evaluated using the GRADE approach for these meta-analyses.

**Table 2:** Meta-analyses results for the Canadian C-spine Rule.

**Main analysis (very low certainty of evidence)** **(10 studies; 35,544 patients; 1213 events)**	
Sensitivity (95% CI)	0.999 (0.976–1)
Specificity (95% CI)	0.188 (0.063–0.443)
Positive LR (95% CI)	1.23 (0.978–1.548)
Negative LR (95% CI)	0.007 (0.001–0.082)
FPR (95% CI)	0.812 (0.557–0.937)
**Subgroup analysis: only fractures evaluated with a CT (low certainty of evidence)** **(3 studies; 10,825 patients; 854 events)**	
Sensitivity (95% CI)	1 (0.996–1)
Specificity (95% CI)	0.02 (0.005–0.072)
Positive LR (95% CI)	1.02 (0.093–1.048)
Negative LR (95% CI)	0.001 (0–0.115)
FPR (95% CI)	0.98 (0.928–0.995)
**Subgroup analysis: ‘cervical spine injury’ as the outcome (very low certainty of evidence)** **(7 studies; 24,719 patients; 359 events)**	
Sensitivity (95% CI)	0.998 (0.924–1)
Specificity (95% CI)	0.398 (0.216–0.613)
Positive LR (95% CI)	1.658 (1.177–2.335)
Negative LR (95% CI)	0.004 (0–0.138)
FPR (95% CI)	0.602 (0.387–0.784)
**Sensitivity analysis: exclusion of studies with a mixed population (very low certainty of evidence)** **(9 studies; 33,102 patients; 875 events)**	
Sensitivity (95% CI)	1 (0.95–1)
Specificity (95% CI)	0.189 (0.056–0.479)
Positive LR (95% CI)	1.234 (0.952–1.598)
Negative LR (95% CI)	0.001 (0–0.14)
FPR (95% CI)	0.811 (0.521–0.944)

LR, Likelihood ratio; FPR, False positive rate.

**Table 3:** GRADE assessments of the meta-analyses pooling the results for the Canadian C-spine Rule.

Meta-analysis	Number of studies (number patients/events)	Factors that may decrease the quality						Factors that may increase the quality		Results			Certainty of evidence
		Phase of investigation	Study limitations	Inconsistency	Indirectness	Imprecision	Publication bias	Moderate or large effect size	Exposure-response gradient	Sensitivity (95% CI)	Specificity (95% CI)	AUC (95% CI)	
Main Analysis (C-spine rule)	10 studies (35,544 patients with 1213 events)	External validation studies	Very serious	Serious	Serious	No	No	Yes for sensitivity	No	0.999 (0.976–1)	0.188 (0.063–0.443)	NA	**Very low**
Subgroup analysis: only fractures evaluated with a CT	3 studies (10,825 patients with 854 events)	External validation studies	Very serious	Serious	No	No	No	Yes for sensitivity	No	1 (0.996–1)	0.02 (0.005–0.072)	NA	**Low**
Subgroup analysis: ‘cervical spine injury’ as the outcome	7 studies (24,719 patients with 359 events)	External validation studies	Very serious	Serious	Serious	No	No	Yes for sensitivity	No	0.998 (0.924–1)	0.398 (0.216–0.613)	NA	**Very low**
Sensitivity analysis: exclusion of studies with a mixed population	9 studies (33,102 patients with 875 events)	External validation studies	Very serious	Serious	Serious	No	No	Yes for sensitivity	No	1 (0.996–1)	0.02 (0.005–0.072)	NA	**Very low**
Discrimination of the C-spine rule	2 studies (6938 patients with 125 events)	External validation studies	Very serious	Serious	Serious	No	No	Yes	No	NA	NA	0.85 (0.72–0.97)	**Very low**

Regarding discrimination, Bandiera et al.^40^ and Ghelichkhani et al.^35^ reported the AUC. The results were pooled in a random effects meta-analysis (2 studies; 6938 patients; 125 events): estimated AUC of 0.850 (95% CI 0.720–0.970; 95% prediction interval 0.630 to 1; very low certainty of the evidence).

#### External validation in adults in out-of-hospital and non-physician-administered contexts

Stiell et al. (2010)^37^ externally validated the Canadian C-spine Rule when applied in adults by nurses in the emergency department. Their study showed a sensitivity of 90.2% (95% CI 76%–95%), a specificity of 43.9% (95% CI 42%–46%), and a negative predictive value of 99.7% (very low certainty of the evidence). Additionally, Vaillancourt et al. (2009)^38^ and Vaillancourt et al. (2023)^39^ conducted external validations of the Canadian C-spine Rule in out-of-hospital settings, applied by paramedics to an adult population, reporting sensitivity ranging from 90.9% to 100% and specificity ranging from 37.7% to 66.5% (very low certainty of the evidence).

#### External validation in children in the paediatric trauma centre setting

Ehrlich et al. (2009)^41^ externally validated the Canadian C-spine Rule in a study involving 125 children aged 10 years or younger who presented at a paediatric trauma centre. The study, which included a total of 7 cervical spine injuries, found the rule to have a sensitivity of 86% and a specificity of 94% (low certainty of the evidence).

### Diagnostic models for traumatic fractures of the cervical spine

#### Development in adults

Seven studies^13^^,^^33^^,^^34^^,^^42^, ^43^, ^44^ developed a total of eight different models for the diagnosis of traumatic cervical spine fractures in adults presenting to an emergency department or a trauma centre. The AUC of the models ranged from 0.65 to 0.91. Two models were evaluated for their calibration, demonstrating good calibration levels as indicated by a plot of the predicted risk score compared to the observed risk in one case^42^ and a Hosmer–Lemeshow test p-value of 0.94 in the other.^13^ The model developed by Inagaki et al. (2018)^45^ was assessed solely using classification measures. It demonstrated a sensitivity of 100% (95% CI: 90.8%–100%), a specificity of 51.9% (95% CI: 48.6%–55.2%), and a negative predictive value of 100% (very low certainty of the evidence).

#### Development in children

Leonard et al. (2011)^46^ developed two models for traumatic cervical fractures in children younger than 16 years old. The models showed a sensitivity of 94% (95% CI 91%–96%) with a specificity of 32% (95% CI 29%–35%; very low certainty of the evidence), and a sensitivity of 92% (95% CI 89%–94%) with a specificity of 35% (95% CI 32%–38%; very low certainty of the evidence).

#### External validation

Inagaki et al. (2018)^45^ also externally validated a previously developed model. Their findings showed a sensitivity of 92.1% (95% CI: 79.2%–97.3%), a specificity of 58.6% (95% CI: 55.4%–61.9%), and a negative predictive value of 99.4% (very low certainty of the evidence). However, the model was not evaluated in terms of discrimination, calibration, or clinical utility.

### Diagnostic models for traumatic fractures of the thoracolumbar spine

Three studies^47^, ^48^, ^49^ developed a total of four different models for the diagnosis of traumatic fractures in the thoracolumbar spine in adults presenting to an emergency department or trauma centre. The sensitivity ranged from 64% to 100%, while the specificity ranged from 29% to 93%. The model developed by Singh et al.^49^ (2011) was also evaluated in terms of discrimination, with a reported AUC of 0.88 (low certainty of the evidence). One of the models developed by Inaba et al. (2015)^48^ was assessed only in terms of discrimination (AUC: 0.81; 95% CI 0.78–0.83; very low certainty of the evidence). No external validation was conducted on these models, and no assessment of calibration and clinical utility was performed during the development phase.

### Diagnostic models for osteoporotic fractures of the thoracolumbar spine

Four studies^50^, ^51^, ^52^, ^53^ developed four different diagnostic models for osteoporotic fractures in the thoracolumbar spine. The study by Ikemoto et al. (2022)^51^ included male and female patients; whereas the remaining three^50^^,^^52^^,^^53^ studies included only females. The AUC for the four models ranged from 0.77 to 0.88. Sensitivity and specificity measures varied between 72.4% and 97.5% for sensitivity, and 72.9%–95% for specificity. The model developed by Khera et al. (2022)^52^ underwent internal validation and demonstrated good calibration, with a calibration slope of 1.0 (low certainty of the evidence). None of the models were externally validated.

### Diagnostic models for all types of fractures of the thoracolumbar and cervical spine

Two studies^54^^,^^55^ developed two different models for all types of thoracolumbar spine fractures in adults presenting to a primary care clinician. The AUC were reported as 0.78 (95% CI 0.69–0.97; very low certainty of the evidence) and 0.83 (95% CI 0.67–1; very low certainty of the evidence). Sensitivity and specificity were presented at various thresholds of the models. Cook et al. (2013) developed a model for assessing all types of cervical spine fractures in a tertiary care setting. The model showed 100% sensitivity (95% CI 100%–100%) and 15% specificity (95% CI 9%–21%) when at least one of the six criteria was met (low certainty of the evidence). Conversely, when all criteria were satisfied, sensitivity dropped to 18% (95% CI 0%–41%), while specificity increased to 100% (95% CI 100%–100%) (low certainty of the evidence). None of the models were externally validated.

## Discussion

Our systematic review aimed to evaluate the performance of multivariable diagnostic prediction models for identifying spinal fractures in people with spinal pain and/or trauma. We identified a total of 17 different models for diagnosing traumatic fractures. One of these (the Canadian C-spine Rule) was externally validated in 10 studies. For osteoporotic fractures, we found four developed models, one of which was internally validated. Additionally, three models were created to diagnose all types of fractures. We did not find any models specifically designed to identify stress fractures.

The Canadian C-spine Rule has been externally validated in ten studies. Our meta-analyses demonstrate excellent sensitivity (0.999; 95% CI 0.976–1), but with very low certainty of the evidence according to the GRADE assessment. A sensitivity close to 1 indicates that the rule is highly effective in identifying nearly all patients with a fracture, minimising false negatives, and therefore making the Canadian C-spine Rule particularly useful when the result is negative, as it can reliably rule out the presence of disease. The AUC is also high (0.850; 95% CI 0.720–0.970), but also with very low certainty. Although the interpretation of AUC values is context-dependent, as highlighted in recent literature, a value above 0.8 is generally considered to reflect good overall discriminative ability.^56^ However, its specificity is extremely poor (0.188; 95% CI 0.063–0.443; very low certainty of the evidence). This low specificity indicates a high proportion of false positives, meaning that many patients without a fracture would still test positive. Therefore, our results indicate that the Canadian C-spine Rule significantly reduces the post-test probability of a fracture following a negative result (negative likelihood ratio: 0.007; 95% CI 0.001–0.082). In fact, a negative likelihood ratio below 0.1 is considered strong evidence to rule out a condition, and the observed value of 0.007 suggests excellent ability to exclude fractures when the test is negative.^57^

Conversely, a positive result does not significantly increase the probability of a fracture, as indicated by a positive likelihood ratio of 1.230 (95% CI 0.978–1.548). The point estimate is close to 1, suggesting that a positive test result only minimally alters the post-test probability. Moreover, the confidence interval includes 1 (the value indicating no change in the post-test probability), implying that we cannot exclude the possibility that a positive result has no diagnostic impact at all. The impact of these findings on clinical practice should be interpreted cautiously due to the very low certainty of the evidence, which reduces our confidence in these estimates. Moreover, the pooled sensitivity and specificity had high heterogeneity, as indicated by the prediction ellipse in Fig. 3. This suggests that the performance of the Canadian C-spine Rule can vary considerably across various settings and populations. At the same time, the prediction interval for the AUC ranged from 0.63 to 1, indicating substantial uncertainty regarding the model's discriminatory ability. This broad range suggests that, while the rule generally performs well, its accuracy may vary considerably across different contexts or subpopulations. From an implementation perspective, such a wide interval implies that the model's performance could differ significantly in new settings.

Therefore, clinicians and decision-makers should interpret the AUC with caution and consider conducting local validation before adopting the rule in practice. It is also important to note that our main meta-analysis included seven studies that raised concerns regarding their applicability because they evaluated the broader outcome of “cervical spinal injury”, which encompasses conditions beyond fractures. This raises questions about the model's specific performance in fractures. Nevertheless, our subgroup analysis, which focused on three studies examining fractures solely through CT imaging, compared to the studies assessing “cervical spinal injury” using various imaging methods, suggests that the issue may not significantly affect the applicability. The negative likelihood ratio was excellent in both groups: 0.001 (95% CI 0–0.115) for fractures alone and 0.004 (95% CI 0–0.138) for the broader outcome. The only observed difference was the wider confidence intervals in the subgroup analyses, likely due to reduced statistical power.

Three studies^37^, ^38^, ^39^ examined the accuracy of the Canadian C-spine Rule when applied by nurses and paramedics. The results revealed lower classification measures, indicating that the rule's accuracy relies on the clinical experience and training of the professional. This emphasises proper training before implementing the rule in non-physician settings. In addition, only one study externally validated the Canadian C-spine Rule in children.^41^ This study did not report confidence intervals for sensitivity and specificity, and the small sample size with only seven fracture events suggests considerable uncertainty in the reported classification values. Consequently, further studies are needed before the Canadian C-spine Rule can be reliably used in paediatric populations.

Among the remaining studies, we found only one externally validated model for identifying cervical traumatic fractures.^45^ However, this model was evaluated with very low certainty of the evidence and has not been assessed for discrimination, calibration, or clinical utility, making it impossible to determine its overall performance. Another model developed by Athinartrattanapong et al. (2021)^42^ for diagnosing cervical traumatic fractures shows promising results, demonstrating good calibration, discrimination, and classification measures. This indicates that an external validation study would be beneficial to confirm its diagnostic accuracy. Similarly, Stiell et al. (2001)^13^ developed a logistic regression model with excellent discrimination and classification values. Although calibration was assessed using the Hosmer–Lemeshow test, which is now discouraged for evaluating model calibration,^21^ the model is a potential candidate for external validation. It is important to note that the studies by Athinartrattanapong et al. (2021)^42^ and Stiell et al. (2001)^13^ were rated as having a very low certainty of the evidence, raising concerns about the validity of their findings. Therefore, while these models may show promise, their high risk of bias highlights the necessity for multiple external validation studies with a low risk of bias before they can be considered for use in clinical practice.

Regarding diagnostic models for traumatic fractures of the thoracolumbar spine, we did not identify any externally validated models. Moreover, none of the developed models appeared sufficiently promising to warrant external validation. This is primarily due to their high risk of bias, lack of internal validation, and absence of key performance measures such as discrimination and calibration. In fact, all models were rated at a very low certainty of the evidence.

Finally, no models have been externally validated for diagnosing osteoporotic fractures and none is therefore ready for use in clinical practice. However, the model developed by Khera et al. (2022)^52^ appears promising for external validation for identifying female osteoporotic fractures. This is supported by its good calibration and discrimination observed after internal validation, suggesting its potential as a valuable tool if confirmed through further low risk of bias studies.

The implications of our findings differ between emergency departments and other clinical settings. In emergency care, the Canadian C-spine Rule, although supported by evidence of very low to low certainty, has demonstrated consistently high sensitivity and may be cautiously considered as a screening tool to rule out traumatic cervical fractures. However, the low specificity and the wide prediction intervals reduce our confidence in its overall accuracy and appropriateness for broad clinical use. In contrast, in non-emergency settings such as primary care or outpatient clinics, where spinal pain presentations are common and the pre-test probability of fracture is lower, our review highlights a substantial evidence gap. No diagnostic models have been externally validated for use in these contexts, underscoring the urgent need for tools that can support safe and efficient decision-making while minimising unnecessary imaging. Importantly, even within emergency settings, we found no validated diagnostic models for traumatic thoracolumbar or osteoporotic fractures.

There is a pressing need to create new diagnostic models for traumatic thoracolumbar fractures and osteoporotic fractures. Future research should also prioritise the external validation of promising diagnostic models, such as the one developed by Khera et al. (2022) for female osteoporotic fractures. Given the very low certainty of evidence supporting the Canadian C-spine Rule, future external validation studies of this model emphasising rigorous study designs with a low risk of bias are needed. Importantly, future studies should also assess model calibration using appropriate methods. In our review, one study evaluated calibration using the Hosmer–Lemeshow test, which has important limitations—most notably, the null hypothesis assumes perfect calibration, and non-significant results are often incorrectly interpreted as evidence of good calibration. Moreover, when the test is statistically significant, it does not provide information on the magnitude or direction of miscalibration. Therefore, future validation studies should incorporate more informative approaches, such as calibration plots, calibration intercepts, and calibration slopes.^18^ Furthermore, developing models that provide individualised probabilities of having a cervical fracture would be beneficial rather than presenting a simple dichotomous outcome. Such models could enhance clinical decision-making by allowing tailored risk assessments for each patient. In addition, expanding research to include underrepresented populations, such as children, and assessing models in diverse clinical settings, such as the use of the Canadian C-spine Rule in out-of-hospital environments, will be essential.

This systematic review has both strengths and limitations. One of its strengths is that it followed rigorous methodological standards, including the prospective registration of a protocol, the use of the CHARMS checklist and PROBAST tool for data extraction and assessing the risk of bias and applicability, and the use of the PRISMA-SRMA checklist for its reporting. Also, it employed a comprehensive search strategy across multiple databases, further enhanced by backward and forward citation tracking. However, it is important to recognise certain limitations. First, we included only studies published in English, Dutch, or Italian, introducing a possible language bias. Second, we used an adapted version of the GRADE framework to assess the certainty of the evidence for prognostic factor studies. Our approach might not be perfect, but currently, there are no definite guidelines for judging the certainty of the evidence for the performance measures of prediction models. Third, we decided to include studies that assessed broader outcomes (i.e., cervical spinal injuries) instead of explicitly focusing on fractures, partially constraining the applicability of our findings. Fourth, we did not pre-specify in our protocol thresholds for what would be considered acceptable or clinically useful levels of predictive performance (e.g., sensitivity, specificity, discrimination, calibration), which may have introduced subjective bias in the interpretation and synthesis of the models’ performance. Fifth, we were only able to perform a meta-analysis for the Canadian C-spine Rule, as there were no multiple studies assessing the other models, thus preventing meaningful data pooling. Sixth, we reported a subgroup analysis for the Canadian C-spine Rule (i.e., studies focused solely on fractures versus those including broader cervical spine injuries), but we did not perform formal statistical tests to compare subgroups due to the methodological challenges of conducting such analyses within a bivariate meta-analytic framework, where sensitivity and specificity are jointly modeled and correlated. Finally, it would have been of interest to conduct a meta-regression analysis to explore the potential association between risk of bias and model performance, as studies with higher risk of bias may overestimate diagnostic accuracy. However, this analysis was not feasible in our review due to the limited number of studies included in the meta-analysis (n = 10) and the lack of variability in the moderator, as all studies were judged to be at high overall risk of bias.

In conclusion, our findings indicate that, while the Canadian C-spine Rule shows potential for screening traumatic cervical fractures, the very low to low certainty of the evidence limits confidence in its accuracy and appropriateness for clinical use. We did not identify any externally validated models suitable for clinical use regarding osteoporotic fractures, traumatic fractures of the thoracolumbar spine, and traumatic fractures of the cervical spine in non-emergency settings. Future research on diagnostic prediction models for spinal fractures should address these gaps.

## Contributors

D.F.: Conceptualisation, Methodology, Study Selection, Data Extraction, Data Analysis, Writing. R.W.: Methodology, Study Selection, Data Extraction, Writing. E.O: Methodology, Writing.

B.K.: Conceptualisation, Methodology, Writing. A.C.: Conceptualisation, Methodology, Study Selection, Data Extraction, Writing. D.F. and R.W. accessed and verified the underlying data.

## Data sharing statement

No new datasets were generated or analysed for this study. All data supporting the findings are derived from previously published studies, which are cited in the manuscript.

## Declaration of interests

A.C. received funding paid to his institution from the ZonMw Huisartsgeneeskunde en Ouderengeneeskunde Programme (The Netherlands), the ZonMw Goed Gebruik Geneesmiddelen Programme (The Netherlands), and the European Horizon 2020 Research and Innovation Programme (European Union). B.K. received funding paid to his institution from the ZonMw Huisartsgeneeskunde en Ouderengeneeskunde Programme (The Netherlands) and the ZonMw Goed Gebruik Geneesmiddelen Programme (The Netherlands).

All other authors declare no conflicts of interest.
