# Supplementary material for: Diagnostic prediction models for spinal fractures in individuals with spinal pain or trauma: a systematic review and meta-analysis
Source: eClinicalMedicine. 2025 Aug 26;88:103456. doi: 10.1016/j.eclinm.2025.103456 (PMC12572814; doi:10.1016/j.eclinm.2025.103456)
Supplement: Supplementary Material 1 [file mmc1.docx]

**SEARCH STRATEGIES FOR PATIENTS WITH SPINAL PAIN**

**Medline**

(exp Spinal Fractures / OR (((spine* OR spinal* OR back* OR burst OR compression* OR sacrum* OR sacral* OR vertebra* OR metasta* OR osteopor* OR trauma* OR predict*) ADJ3 fracture*) OR chance-fracture*).ab,ti,kw. OR (fracture*).ti.) AND (exp Back Pain/ OR Neck Pain/ OR (((spine* OR spinal* OR back OR sacroiliac* OR discogenic* OR thoracic* OR neck OR cervical* OR vertebra* OR lumbar*) ADJ3 pain*) OR backache* OR back-ache* OR backpain* OR dorsalgia* OR cervicalgia OR lumbago*).ab,ti,kw.) AND ("Sensitivity and Specificity"/ OR (sensitivit* OR specificit*).ab,ti,kw. OR (Validat* OR Predict*.ti. OR Rule*) OR (Predict* AND (Outcome* OR Risk* OR Model*)) OR ((History OR Variable* OR Criteria OR Scor* OR Characteristic* OR Finding* OR Factor*) AND (Predict* OR Model* OR Decision* OR Identif* OR Prognos*)) OR (Decision* AND (Model* OR Clinical* OR Models, Statistical / OR Logistic Models)) OR (Prognostic AND (History OR Variable* OR Criteria OR Scor* OR Characteristic* OR Finding* OR Factor* OR Model*)) OR Stratification OR ROC Curve / OR Discrimination OR Discriminate OR c-statistic OR c-statistic OR Area-under-the-curve OR AUC OR Calibration OR Indices OR Algorithm OR Multivariable OR (Canadian-c-spine-rule* OR nexus OR National-Emergency-XRadiography-Utilization-Study).ab,ti,kw.) NOT (exp animals/ NOT humans/) AND (english.la. OR dutch.la. OR Italian.la) NOT (Systematic Review / OR Meta-Analysis / OR ((systematic* ADJ3 review*) OR (meta-analys*)).ti.) NOT (Case Reports / OR (case-stud* OR case-report* OR case-ser*).ti.)

**Embase**

('spine fracture'/exp OR (((spine* OR spinal* OR back* OR burst OR compression* OR sacrum* OR sacral* OR vertebra* OR metasta* OR osteopor* OR trauma* OR predict*) NEAR/3 fracture*) OR chance-fracture*):ab,ti,kw OR (fracture*):ti) AND ('spinal pain'/de OR backache/exp OR 'neck pain'/exp OR (((spine* OR spinal* OR back OR sacroiliac* OR discogenic* OR thoracic* OR neck OR cervical* OR vertebra* OR lumbar*) NEAR/3 pain*) OR backache* OR back-ache* OR backpain* OR dorsalgia* OR cervicalgia OR lumbago*):ab,ti,kw) AND ('sensitivity and specificity'/de OR (sensitivit* OR specificit*):Ab,ti,kw OR (Validat* OR Predict*:ti OR Rule*) OR (Predict* AND (Outcome* OR Risk* OR Model*)) OR ((History OR Variable* OR Criteria OR Scor* OR Characteristic* OR Finding* OR Factor*) AND (Predict* OR Model* OR Decision* OR Identif* OR Prognos*)) OR (Decision* AND (Model* OR Clinical* OR 'statistical model'/de)) OR (Prognostic AND (History OR Variable* OR Criteria OR Scor* OR Characteristic* OR Finding* OR Factor* OR Model*)) OR Stratification OR 'receiver-operating-characteristic'/de OR Discrimination OR Discriminate OR c-statistic OR c-statistic OR Area-under-the-curve OR AUC OR Calibration OR Indices OR Algorithm OR Multivariable OR 'canadian c spine rule'/de OR (Canadian-c-spine-rule* OR nexus OR National-Emergency-XRadiography-Utilization-Study):Ab,ti,kw) NOT [conference abstract]/lim NOT ([animals]/lim NOT [humans]/lim) AND ([english]/lim OR [dutch]/lim OR [italian]/lim) NOT ('systematic review'/de OR 'meta analysis'/de OR ((systematic* NEAR/3 review*) OR (meta-analys*)):ti) NOT ('case report'/de OR 'case study'/de OR (case-stud* OR case-report* OR case-ser*):ti)

**Web of science**

(TS=(((spine* OR spinal* OR back* OR burst OR compression* OR sacrum* OR sacral* OR vertebra* OR metasta* OR osteopor* OR trauma* OR predict*) NEAR/2 fracture*) OR chance-fracture*) OR TI=(fracture*)) AND TS=((((spine* OR spinal* OR back OR sacroiliac* OR discogenic* OR thoracic* OR neck OR cervical* OR vertebra* OR lumbar*) NEAR/2 pain*) OR backache* OR back-ache* OR backpain* OR dorsalgia* OR cervicalgia OR lumbago*)) AND TS=((sensitivit* OR specificit*) OR (Validat* OR Rule*) OR (Predict* AND (Outcome* OR Risk* OR Model*)) OR ((History OR Variable* OR Criteria OR Scor* OR Characteristic* OR Finding* OR Factor*) AND (Predict* OR Model* OR Decision* OR Identif* OR Prognos*)) OR (Decision* AND (Model* OR Clinical*)) OR (Prognostic AND (History OR Variable* OR Criteria OR Scor* OR Characteristic* OR Finding* OR Factor* OR Model*)) OR Stratification OR Discrimination OR Discriminate OR c-statistic OR c-statistic OR Area-under-the-curve OR AUC OR Calibration OR Indices OR Algorithm OR Multivariable OR (Canadian-c-spine-rule* OR nexus OR National-Emergency-XRadiography-Utilization-Study)) NOT TI=(((systematic* NEAR/2 review*) OR (meta-analys*))) NOT TI=((case-stud* OR case-report* OR case-ser*)) NOT DT=(Meeting Abstract OR Meeting Summary) AND LA=(English OR dutch OR italian)

**SEARCH STRATEGIES FOR PATIENTS WITH SPINAL TRAUMA**

**Medline**

(Spinal Fractures / OR exp * Spine/in OR * Spinal Injuries/ OR (((spine* OR spinal* OR vertebra*) ADJ3 (trauma* OR fracture*)) OR chance-fracture*).ab,ti,kw. OR ((spine OR spinal) AND (trauma* OR fracture*)).ti.) AND (* "Sensitivity and Specificity"/ OR * Predictive Value of Tests / OR * Reproducibility of Results / OR * Decision Support Techniques*/ OR * Triage/ OR ((Predictive Value of Tests / OR Decision Support Techniques/ OR Triage/) AND ("Sensitivity and Specificity"/ OR Validation Study / OR Reproducibility of Results /)) OR (((scoring OR rule*) ADJ6 (validat* OR validit* OR sensitive* OR specific* OR evaluat* OR reproducib*)) OR ((predict* OR forecast*) ADJ3 (model* OR simulat*))).ab,ti,kw. OR (scoring-system* OR rule* OR validat* OR validit* OR sensitive* OR specific* OR model* OR simulation* OR reproducib* OR ((predict* OR score* OR scoring* OR forecast*) AND fracture*)).ti.) NOT (exp animals/ NOT humans/) AND (english.la. OR dutch.la. OR Italian.la.) NOT (Systematic Review / OR Meta-Analysis / OR ((systematic* ADJ3 review*) OR (meta-analys*)).ti.) NOT (Case Reports / OR (case-stud* OR case-report* OR case-ser*).ti.)

**Embase**

('spine fracture'/exp OR 'spine injury'/mj OR (((spine* OR spinal* OR vertebra*) NEAR/3 (trauma* OR fracture*)) OR chance-fracture*):ab,ti,kw OR ((spine OR spinal) AND (trauma* OR fracture*)):ti) AND ('predictive model'/de OR 'scoring system'/mj OR 'sensitivity and specificity'/mj OR 'validation process'/mj OR 'validation study'/mj OR 'predictive value'/mj OR reproducibility/mj OR (('scoring system'/de OR 'predictive value'/de) AND ('sensitivity and specificity'/de OR 'validation process'/de OR 'validation study'/de OR reproducibility/de)) OR (((scoring OR rule*) NEAR/6 (validat* OR validit* OR sensitive* OR specific* OR evaluat* OR reproducib*)) OR ((predict* OR forecast*) NEAR/3 (model* OR simulat*))):Ab,ti,kw OR (scoring-system* OR rule* OR validat* OR validit* OR sensitive* OR specific* OR model* OR simulation* OR reproducib* OR ((predict* OR score* OR scoring* OR forecast*) AND fracture*)):ti) NOT [conference abstract]/lim NOT ([animals]/lim NOT [humans]/lim) AND ([english]/lim OR [dutch]/lim OR [italian]/lim) NOT ('systematic review'/de OR 'meta analysis'/de OR ((systematic* NEAR/3 review*) OR (meta-analys*)):ti) NOT ('case report'/de OR 'case study'/de OR (case-stud* OR case-report* OR case-ser*):ti)

**Web of science**

(TS=(((spine* OR spinal* OR vertebra*) NEAR/2 (trauma* OR fracture*)) OR chance-fracture*) OR TI=((spine OR spinal) AND (trauma* OR fracture*))) AND (TS=(((scoring OR rule*) NEAR/5 (validat* OR validit* OR sensitive* OR specific* OR evaluat* OR reproducib*)) OR ((predict* OR forecast*) NEAR/2 (model* OR simulat*))) OR TI=(scoring-system* OR rule* OR validat* OR validit* OR sensitive* OR specific* OR model* OR simulation* OR reproducib* OR ((predict* OR score* OR scoring* OR forecast*) AND fracture*))) NOT TI=(((systematic* NEAR/2 review*) OR (meta-analys*))) NOT TI=((case-stud* OR case-report* OR case-ser*)) NOT DT=(Meeting Abstract OR Meeting Summary) AND LA=(English OR dutch OR italian)
