# Supplementary material for: Diagnostic prediction models for spinal fractures in individuals with spinal pain or trauma: a systematic review and meta-analysis
Source: eClinicalMedicine. 2025 Aug 26;88:103456. doi: 10.1016/j.eclinm.2025.103456 (PMC12572814; doi:10.1016/j.eclinm.2025.103456)
Supplement: Supplementary Material 2 [file mmc2.docx]

External validation studies started with a grading of a “high quality of evidence”, whereas development studies started with a grading of a “moderate quality of evidence”. The quality of the evidence was downgraded in case of study limitations (serious limitations when most evidence is from studies with an overall moderate or unclear risk of bias, or very serious limitations when most evidence is from studies with an overall high risk of bias), inconsistency, indirectness, imprecision, or publication bias. Downgrading for inconsistency was applied when substantial heterogeneity was observed in the bivariate meta-analysis, typically reflected by wide 95% prediction intervals or visually large dispersion in the summary ROC space. While we did not rely on a fixed threshold, heterogeneity was judged as “serious” when the 95% prediction region included values with markedly different clinical implications. Imprecision was judged based on the width of the 95% confidence intervals around the pooled point estimates. For sensitivity, although the point estimates were often high (e.g., >0.9), we downgraded when confidence intervals were wide enough to include values that could compromise clinical utility (e.g., lower bounds <0.85). The quality of the evidence was upgraded in case of a moderate or large predictive ability (i.e., AUC > 0.7, sensitivity > 0.9 or specificity > 0.7) or an exposure-response gradient.
