# Supplementary material for: Diagnostic prediction models for spinal fractures in individuals with spinal pain or trauma: a systematic review and meta-analysis
Source: eClinicalMedicine. 2025 Aug 26;88:103456. doi: 10.1016/j.eclinm.2025.103456 (PMC12572814; doi:10.1016/j.eclinm.2025.103456)
Supplement: Supplementary Material 3 [file mmc3.docx]

| **First author (year)** | **Country** | **Setting** | **Study type** | **Cohort type** | **Spine location** | **Outcome** | **Outcome definition** |
| --- | --- | --- | --- | --- | --- | --- | --- |
| Athinartrattanapong (2021) | Thailand | Emergency department | D | Cross-sectional  study | Cervical spine | Traumatic cervical spinal injury | Cervical spine fracture, subluxation, dislocation, or traumatic spondylolisthesis confirmed by CT scan |
| Bandiera (2003) | Canada | Emergency department | V | Cross-sectional  study | Cervical spine | Traumatic cervical spinal injury | Clinically significant spine injuries confirmed by X-ray, CT scan, or nurse-led telephone interview 14 days post-discharge; non-clinically significant injuries included isolated osteophyte avulsion, transverse or spinous process fractures, and compression fractures involving less than 25% of the vertebral body |
| Bub (2005) | United States | Trauma center | D + IV | Case – control study | Cervical spine | Traumatic fracture | Cervical spine fracture confirmed through medical records and unspecified radiologic reports |
| Caltili (2017) | Turkey | Emergency department | V | Case – control study | Cervical spine | Traumatic fracture | Traumatic cervical spine fracture confirmed by CT |
| Clark (2016) | United Kingdom | Primary (around 80% of the included patients) and secondary care | D | Case – control study | Thoracic spine | Osteoporotic fracture | Vertebral fracture identified on thoracic spine X-rays using an algorithm-based qualitative method |
| Coffrey (2015) | United Kingdom | Emergency department | V | Cross-sectional  study | Cervical spine | Traumatic cervical spine injury | Cervical spine injury confirmed by X-ray |
| Cook (2013) | United States | Tertiary care (Department of surgery) | D | Retrospective cohort study | Cervical spine | Fracture (all types) | Fracture diagnosis by a board-certified orthopedic surgeon using MRI, X-ray, and/or CT |
| Duane (2011) | United States | Trauma center | D + V | Cross-sectional  study | Cervical spine | Traumatic fracture | Fracture confirmed by CT |
| Duane (2013) | United States | Trauma center | D + V | Cross-sectional  study | Cervical spine | Traumatic fracture | Fracture confirmed by CT |
| Ehrlich (2009) | United States | Pediatric trauma center | V | Retrospective cohort study | Cervical spine | Traumatic cervical spine injury | Clinically significant spine injury defined as fracture, dislocation, or ligamentous instability confirmed by X-ray or CT |
| Engelbart (2021) | United States | Trauma center | D + IV | Cross-sectional  study | Cervical spine | Traumatic cervical spine injury | Cervical spine injury confirmed by CT, X-ray, or medical chart review for subsequent visits or admissions for cervical spine symptoms after the initial ground-level fall |
| Enthoven (2016) | The Netherlands | General practices | D | Prospective cohort study | Thoracic and lumbar spine | Fracture (all types) | Spinal fracture diagnosis at 1-year follow-up using general practitioner registry data; all patients underwent lumbar/thoracic spine X-ray |
| Ghelichkhani (2021) | Iran | Emergency department | V | Cross-sectional  study | Cervical spine | Traumatic cervical spine injury | Clinically significant spine injury, including fracture-dislocation and vertebral instability, confirmed by X-ray, CT, or MRI |
| Henschke (2009) | Australia | Primary care | D | Prospective cohort study | Lumbar spine | Fracture (all types) | Participants were contacted at 6 weeks, 3 months, and 12 months to report any serious diagnoses of low back pain. A study rheumatologist examined those with a potential spinal fracture |
| Hercz (2019) | United States | Emergency department | D | Retrospective cohort study | Thoracolumbar spine | Traumatic thoracolumbar spine injury | Acute thoracolumbar spine injury confirmed by CT, MRI, or X-ray, including those requiring orthopedic evaluation, bracing, or surgical stabilization |
| Ikemoto (2022) | Japan | Secondary care | D | Cross – sectional study | Lower thoracic and lumbar spine | Osteoporotic fracture | Presence of cortical line discontinuity on X-ray or CT or abnormal vertebral body intensity on MRI |
| Inaba (2015) | United States | Trauma centers | D | Cross-sectional  study | Thoracolumbar spine | Traumatic thoracolumbar spine injury | Clinically significant thoracolumbar fracture requiring orthosis or surgical stabilization; isolated transverse and spinous process fractures were deemed insignificant |
| Inagaki (2018) | Japan | Emergency department | D + V | Cross-sectional study | Cervical spine | Traumatic cervical spine injury | Cervical spine injury (fracture or dislocation) detected by CT; patients without CT were followed for 14 days to identify missed injuries |
| Khera (2022) | United Kingdom | Primary care | D + IV | Cross – sectional study | Thoracic and lumbar spine | Osteoporotic fracture | Fracture evaluated on lateral X-ray using an algorithm-based qualitative method |
| Leonard (2011) | United States | Pediatric Emergency Department | D + IV | Case – control study | Cervical spine | Traumatic cervical spine injury | Children with ICD-9 codes for cervical spine injury, including vertebrae, ligament, or spinal cord injuries, and spinal cord injuries without radiographic findings |
| Roux (2007) | France | Not clear | D | Cross – sectional study | Thoracic and lumbar spine (from T4 to L5) | Osteoporotic fracture | Fracture confirmed using three lateral and anteroposterior spine X-rays, defined as grade >1 on Genant’s semiquantitative method |
| Singh (2011) | Australia | Emergency Department | D | Case – control study | Thoracic spine | Traumatic fracture | Fracture confirmed by X-ray or CT |
| Stiell (2001) | Canada | Emergency Department | D + IV | Cross – sectional study | Cervical spine | Traumatic cervical spine injury | Clinically significant cervical spine injury (fracture, dislocation, or ligamentous instability) confirmed by X-ray or CT; non-clinically significant injuries included isolated avulsion fractures, transverse process fractures not involving a facet joint or lamina, and <25% vertebral compression fractures. Patients without imaging were interviewed, and those with mild symptoms and no functional limitations were deemed injury-free; others were recalled for clinical assessment and imaging |
| Stiell (2003) | Canada | Emergency Department | V | Cross-sectional  study | Cervical spine | Traumatic cervical spine injury | Clinically significant cervical spine injury (fracture, dislocation, or ligamentous instability) confirmed by X-ray or CT; non-clinically significant injuries included isolated avulsion fractures, transverse process fractures not involving a facet joint or lamina, and <25% vertebral compression fractures. Patients without imaging were interviewed, and those with mild symptoms and no functional limitations were deemed injury-free; others were recalled for clinical assessment and imaging |
| Stiell (2010) | Canada | Emergency Departments | V | Cross-sectional  study | Cervical spine | Traumatic cervical spine injury | Clinically significant cervical spine injury (fracture, dislocation, or ligamentous instability) confirmed by X-ray or CT; non-clinically significant injuries included isolated avulsion fractures, transverse process fractures not involving a facet joint or lamina, and <25% vertebral compression fractures. For patients without imaging, emergency department visit logs were monitored for 30 days to track return visits |
| Vaillancourt (2009) | Canada | Out-of-hospital | V | Cross-sectional  study | Cervical spine | Traumatic cervical spine injury | Cervical spine injury, defined as any fracture, dislocation, or ligamentous instability confirmed by imaging. Isolated avulsion fractures, transverse process fractures not involving the facet joint, spinous process fractures not involving the lamina, and <25% vertebral compression fractures were excluded. Patients without imaging were contacted within 14 days; those with mild symptoms, no collar use, and normal activity were classified as injury-free. Others were recalled for reassessment and imaging |
| Vaillancourt (2023) | Canada | Out-of-hospital | V | Cross-sectional  study | Cervical spine | Traumatic cervical spine injury | Cervical spine injury, defined as any fracture, dislocation, or ligamentous instability confirmed by imaging. Isolated avulsion fractures, transverse process fractures not involving the facet joint, spinous process fractures not involving the lamina, and <25% vertebral compression fractures were excluded. Patients without imaging were contacted within 14 days; those with mild symptoms, no collar use, and normal activity were classified as injury-free. Others were recalled for reassessment and imaging |
