# Supplementary material for: Diagnostic prediction models for spinal fractures in individuals with spinal pain or trauma: a systematic review and meta-analysis
Source: eClinicalMedicine. 2025 Aug 26;88:103456. doi: 10.1016/j.eclinm.2025.103456 (PMC12572814; doi:10.1016/j.eclinm.2025.103456)
Supplement: Supplementary Material 4 [file mmc4.docx]

| **First author (year)** | **Inclusion criteria** | **Exclusion criteria** |
| --- | --- | --- |
| Athinartrattanapong (2021) | - Aged 16 years or older  - Traumatic neck injury assessed by CT at the emergency department | Not reported |
| Bandiera (2003) | - Adults who were ambulatory or immobilized  - Hemodynamically stable and alert (Glasgow Coma Scale score of 15)  - Neck pain from any mechanism of injury or no neck pain but visible injury above the clavicles, non-ambulatory status, and a dangerous mechanism of injury | - Age younger than 16 years  - No neck pain or visible injury above the clavicle  - Glasgow Coma Scale score less than 15  - Unstable vital signs  - Time of injury more than 48 hours before assessment  - Penetrating trauma  - Acute paralysis  - Known vertebral disease (e.g., ankylosing spondylitis, rheumatoid arthritis, spinal stenosis, previous cervical surgery)  - Pregnancy |
| Bub (2005) | Cases:  - Aged 65 years or older  - Blunt trauma patients with cervical spine fracture confirmed on medical records and radiologic reports  Controls:  - Aged 65 years or older  - Blunt trauma patients without cervical spine fracture | - Penetrating trauma  - Death prior to imaging  - Transfer from another institution |
| Caltili (2017) | Cases:  - Following trauma  - Positive traumatic spinal fracture confirmed by CT  Controls:  - Following trauma  - Negative traumatic spinal fracture | Not reported |
| Clark (2016) | Cases:  - Aged 60 years or older  - Female  - Thoracic radiographs performed within the previous 3 months  Controls:  - No vertebral fracture on thoracic radiographs | - Spinal surgery  - Metastases identified on radiographs |
| Coffrey (2015) | - Aged 16 years or older  - Neck pain following acute blunt trauma to the head and/or neck  - No neck pain but non-ambulatory with evidence of injury above the clavicle  - Alert and stable (Glasgow Coma Score >15 with normal vital signs)  - Injury sustained within the previous 48 hours | - Major trauma  - Penetrating trauma  - Acute paralysis or paresis  - Vertebral disease  - Returned for reassessment  - Pregnancy |
| Cook (2013) | - Assessed in a spinal center as possible candidates for surgery  - Imaging-supported diagnosis | - Variables of interest were missing |
| Duane (2011) | - Aged older than 16 years  - Blunt trauma resulting in trauma team activation | Not reported |
| Duane (2013) | - Aged older than 16 years  - Blunt trauma resulting in trauma team activation | Not reported |
| Ehrlich (2009) | - Aged 10 years or younger  - Following a traumatic event with neck tenderness  - Neurological deficits  - Abnormal Glasgow Coma Score  - Distracting pain from another injury | Not reported |
| Engelbart (2021) | - Aged 65 years or older  - Ground-level falls  - Presented to a hospital trauma facility | - Fall from more than three steps or as part of another traumatic event, such as a motor vehicle accident  - Arrival to the institution more than 48 hours after the fall  - Insufficient data to determine the nature of the fall (ground-level, stairs, or height) |
| Enthoven (2016) | - Aged older than 55 years  - Back pain with no episodes in the previous 6 months  - Consulting a general practitioner for a new episode of back pain | - Cognitive impairment preventing completion of questionnaires  - Inability to read and write in Dutch  - Unable to undergo physical examination (e.g., wheelchair users) |
| Ghelichkhani (2021) | - Aged older than 18 years  - Clinical signs and symptoms of cervical spine injury  - Referred to the emergency department as trauma patients | - Penetrating injury  - Glasgow Coma Scale less than 15  - Acute paralysis  - Known vertebral diseases  - Previous cervical spine injury  - Pregnancy |
| Henschke (2009) | - Aged at least 14 years  - Acute low back pain lasting more than 24 hours but less than 6 weeks, preceded by at least 1 month without back pain  - Pain localized between T12 and the buttock crease, with or without referred pain beyond this region  - Presented to a primary care provider for a first-time consultation | - Serious pathology diagnosed before consultation  - Serious pathology considered the cause of the current episode of low back pain |
| Hercz (2019) | - Aged 18 years or older  - Presented to a major academic center emergency department  - Mild to moderate blunt traumatic injuries  - No prehospital activation of the surgical trauma team | - Lack of dedicated imaging and interpretation of the thoracic, lumbosacral, or thoracolumbar spine  - Previous treatment at an outside hospital for the same injury  - Coexisting conditions preventing history acquisition or examination (e.g., altered mentation from intoxication or other causes)  - Chronic focal neurologic deficit  - Injury chronicity of more than 7 days  - Left prior to complete evaluation |
| Ikemoto (2022) | - Aged 65 years or older  - Acute low back pain within the last 4 weeks | - Radiating pain in one or both legs  - Presence or history of major neurological disorders (e.g., post-stroke or Parkinson’s disease)  - High-energy trauma  - Ongoing malignant disease  - Pyogenic spondylitis  - Dementia |
| Inaba (2015) | - Aged 15 years or older  - Blunt trauma at the time of initial trauma center assessment | - Glasgow Coma Scale score less than 15  - Intoxication  - Painful distracting injury  - Existing paraplegia or tetraplegia  - Concurrent cervical spine injury causing a neurologic deficit  - Initial surgical team assessment more than 24 hours after injury |
| Inagaki (2018) | - Aged older than 16 years  - Head or neck trauma  - Transported to the emergency department by ambulance | - Penetrating neck injury |
| Khera (2022) | - Women aged 65 years or older  - Self-reported episode of back pain in the previous 4 months | Not reported |
| Leonard (2011) | Cases:  - Children younger than 16 years  - Underwent cervical spine radiography after blunt trauma  Controls:  - Selected within 1 year of the assigned case  - Three control groups: random control, mechanism of injury control (matched to cases by age and mechanism of injury), and emergency medical services control (matched to cases by age and out-of-hospital care) | Not reported |
| Roux (2007) | - Ambulatory postmenopausal women aged 65–85 years  - Consultation due to back pain (thoracic or lumbar pain with visual analog scale > 40 mm)  - Osteoporotic based on bone mineral density measured by dual-energy X-ray absorptiometry of the spine, femoral neck, or total femur | - No bisphosphonates, selective estrogen receptor modulators, or hormone replacement therapy for at least 3 months before inclusion |
| Singh (2011) | Cases:  - Aged 15 years or older  - Presented to the emergency department following trauma  - Sustained any type of thoracic spine fracture  Controls:  - Presented to the emergency department following trauma  - Did not sustain a thoracic spine fracture | - Medical records were unavailable |
| Stiell (2001) | - Adults presenting to the emergency department after blunt head or neck trauma at risk for cervical spine injury  - Neck pain from injury mechanism and all of the following: visible injury above the clavicles, not ambulatory, dangerous injury mechanism  - Alert with a Glasgow Coma Scale score of 15  - Stable with normal vital signs | - Younger than 16 years old  - Minor injuries, such as a simple laceration  - Injury occurred more than 48 hours previously  - Penetrating trauma  - Acute paralysis  - Vertebral disease  - Returned for reassessment for the same injury  - Pregnancy |
| Stiell (2003) | - Adults presenting to the emergency department after blunt head or neck trauma at risk for cervical spine injury  - Neck pain from injury mechanism and all of the following: visible injury above the clavicles, not ambulatory, dangerous injury mechanism  - Alert with a Glasgow Coma Scale score of 15  - Stable with normal vital signs | - Younger than 16 years old  - Minor injuries, such as a simple laceration  - Injury occurred more than 48 hours previously  - Penetrating trauma  - Acute paralysis  - Vertebral disease  - Returned for reassessment for the same injury  - Pregnancy |
| Stiell (2010) | - Adults presenting to the emergency department after blunt head or neck trauma  - Posterior neck pain or cervical spine immobilization upon arrival by ambulance  - Alert with a Glasgow Coma Scale score of 15  - Normal vital signs | - Younger than 16 years old  - Minor injuries, such as a simple laceration  - Injury occurred more than 48 hours previously  - Penetrating trauma  - Acute paralysis  - Vertebral disease  - Returned for reassessment for the same injury |
| Vaillancourt (2009) | - Posterior neck pain from any blunt mechanism  - No neck pain but visible injury above the clavicles  - Glasgow Coma Scale score of 15  - Normal vital signs as defined by the Revised Trauma Score (systolic blood pressure ≥ 90 mm Hg and respiratory rate between 10 and 24 breaths per minute upon arrival)  - Willingly follows commands and is not agitated  - Transported by ambulance to local hospitals after sustaining acute blunt trauma (injury within the past 8 hours) | - Age below 16 years  - Penetrating trauma to the neck  - Acute paralysis  - Known vertebral disease |
| Vaillancourt (2023) | - Low-risk trauma patients  - Injury occurred within 4 hours prior to paramedic assessment  - Alert with a Glasgow Coma Scale score of 15  - Stable with systolic blood pressure ≥ 90 mmHg and respiratory rate between 10 and 24 breaths per minute | - Age below 16 years  - Penetrating trauma to the neck  - Acute paralysis  - Known vertebral disease  - Referred from another hospital and only required inter-facility transport. |
