# Supplementary material for: Diagnostic prediction models for spinal fractures in individuals with spinal pain or trauma: a systematic review and meta-analysis
Source: eClinicalMedicine. 2025 Aug 26;88:103456. doi: 10.1016/j.eclinm.2025.103456 (PMC12572814; doi:10.1016/j.eclinm.2025.103456)
Supplement: Supplementary Material 5 [file mmc5.docx]

| **First author (year)** | **Sample size/number of events/methods of sample size calculation** | **Age** | **Gender** | **Type of trauma** | **Pain at baseline** | **Time since the event/start of the symptoms** |
| --- | --- | --- | --- | --- | --- | --- |
| Athinartrattanapong (2021) | 375 (29 cervical spine injuries and 346 non-cervical spine injuries)  Sample size was calculated via a one-sample comparison of proportions, with an alpha of 0.05 (one side), power of 0.8, n2/n1 of 17, and an incidence rate of cervical fracture in the emergency department of 5.88% | Age ≥ 65 years:  Cases: 11  Non-cases: 124 | Cases: 13 males (44.8%)  Non-cases: 212 males (61.3%) | Motor vehicle collision:  Cases: 7  Non-cases: 87  Pedestrian injury:  Cases: 2  Non-cases: 16  Assault/violence: Cases: 0  Non-cases: 29  Falling to the ground:  Cases 4  Non-cases: 119  Fall from a height:  Cases: 6  Non-cases: 21 | Not reported | Not reported |
| Bandiera (2003) | 6265/64 cervical spine injury | Mean 36.6 (SD 16) years | 3.177 males (50.6%) | - Motor vehicle crash: 4,235 (67.6%)  - Fall: 870 (13.9%)  - Pedestrian struck: 194 (3.1%)  - Assault: 213 (3.4%)  - Head struck by object: 209 (3.3%)  - Sports: 182 (2.9%  - Bicycle: 160 (2.6%)  - Axial load: 119 (1.9%)  - Motorcycle: 48 (0.8%)  - Motorized recreational vehicle: 29 (0.5%)  - Other: 6 (0.1%) | 5744 (91.7%) | Not reported |
| Bub (2005) | 210 (103 fractures and 107 non-fractures) | Cases: mean 77 (range 65-102) years  Controls: mean 76 (range 65-101) years | 117 males (55.7%) | Cases:  - High-speed motor vehicle crash (≥ 30 mph): 49 (47%)  - Low or uncertain speed: 9 (9%)  - Fall from standing or sitting height: 11 (11%)  - From height greater than standing: 20 (19%)  - Found down: 6(6%)  - Car striking pedestrian: 3(3%)  - Assaulted: 0  - Airplane crash: 2 (2%)  - Bicycle accident: 2 (2%)  - Skiing accident: 1 91%)  - Fall from moving vehicle: 0 | Not reported | Not reported |
| Caltili (2017) | 2442 patients (338 fractures and 2104 non-fractures) | - 0-9 years: 162 (7%)  - 10-19 years: 283 (12%)  - 20-29 years: 577 (24%)  - 30-39 years: 443 (18%)  - 40-49 years: 333 (14%)  - 50-64 years: 226 (9%)  - ≥ 65 years: 418 (17%) | 1566 males (64%) | Not reported | 1851 (76%) had pain at admission | Not reported |
| Clark (2016) | 197 (64 fractures and 133 non-fractures) | Cases: mean 76.9 (IQR 71.2 to 83.5) years  Controls: mean 71.7 (IQR 67.0 to 78.0) years | All females by inclusion criteria | Not reported | Cases: mean 7.7 on the 0-10 VAS  Controls: mean 7.6 on the 0-10 VAS | Duration of pain:  - “Few days to weeks”: 28 (46.7%)  - “Months to years”: 32 (53.3%)  - “Few days to weeks”: 30 (24.6%)  - “Months to years”: 92 (75.4%) |
| Coffrey (2015) | 1420/8 cervical spine injuries | Not reported | Not reported | Not reported | Not reported | Not reported |
| Cook (2013) | 162/11 fractures | Age ≤ 33: 9 patients (5%) | 79 males (49%) | Not reported | Not reported | Duration of symptoms < 6 weeks: 13 patients (8%) |
| Duane (2011) | 3201/192 fractures | Fracture group: mean 42.7 (SD 19.0) years  Non-fracture group: mean 37.8 (SD 17.5) years | 2051 males (64.1%) | Not reported | Not reported | Not reported |
| Duane (2013) | 5182/324 fractures | Fracture group: mean 43.9 (SD 18.8) years  Non-fracture group: mean 38.4 (SD 17.5) years | 3293 males (63.5%) | Not reported | Not reported | Not reported |
| Ehrlich (2009) | 125/7 cervical spine injuries | Mean 4.3 (SD 3.1) | 72 males (57%) | Not reported | Not reported | Not reported |
| Engelbart (2021) | Training set: 707/75 cervical spine injuries  Validation set: 1605/178 cervical spine injuries | Training set: mean 81.3 (SD 8.4) years  Validation set: mean 80.9 (SD 8.4) years | Training set: 273 (38.6%) males  Validation set: 631 (39.3%) males | Training set:  - Fall from standing: 625 (88.4%)  - Fall from a chair: 43 (6.1%)  - Fall from bed: 39 (5.5%)  Validation set:  - Fall from standing: 1455 (90.7%)  - Fall from chair: 73 (4.5%)  - Fall from bed: 77 (4.8%) | Not reported | Not reported |
| Enthoven (2016) | 669/33 fractures | Mean 66 (SD 7.7) years | 269 males (40%) | Not reported | NRS 5 (SD 2.7) | Duration of back pain > 3 months: 154 (23%) |
| Ghelichkhani (2021) | 673/61 cervical spine injuries  Sample size was calculated based on 95% sensitivity, 10% prevalence, 0.05 marginal error | Mean 34.3 (SD 19.4) years | 466 males (69.2%) | - High speed MVC/rollover/ejection: 259 (38.7%)  - Pedestrian to car: 114 (22.1%)  - Fall from ≥ 3 ft (0.9 m)/5 stairs: 78 (11.7%)  - Bicycle collision: 32 (4.8%)  - Simple rearend motor vehicle collision: 186 (27.8%) | Not reported | Not reported |
| Henschke (2009) | 1172/8 fractures | Mean 43.97 (SD 15.1) years | 626 males (53.4%) | Not reported | Median 4 (range 1 – 6) | - Less than 1 week: 696 (59.4%)  - 1 – 2 weeks: 145 (12.4%)  - 2 – 3 weeks: 174 (14.8%)  - 3 – 4 weeks: 73 (6.2%)  - 4 – 5 weeks: 30 (2.6%)  - 5 – 6 weeks: 54 (4.6%) |
| Hercz (2019) | 1049/36 thoracolumbar spine injuries | Median 46 (range 33-57) years | 561 males 53.4% | - Fall: 463 (44.1%)  - Motor-vehicle collision: 423 (40.3%)  - Pedestrian, bicycle, or motorcycle vs. motor-vehicle collision: 65 (6.2%)  - Assault: 59 (5.6%) | Presence of back pain: 1009 (96.2%) | More than 24 hours from trauma to presentation: 189 (18%) |
| Ikemoto (2022) | 80/40 fractures | Patients with a fracture: mean 79.5 (SD 8.3) years  Patients without a fracture: mean 76.7 (SD 7.6) years | 27 males (34%) | Not reported | Patients with a fracture: median 7.0 (IQR 3.0)  Patients without a fracture: median 5.0 (IQR 2.0) | Patients with a fracture: median 7.5 days (IQR 11.0)  Patients without a fracture: median 7.0 days (IQR 8.75) |
| Inaba (2015) | 3065/264 thoracolumbar spine injuries  Sample size calculation based on a target of 98% sensitivity | Mean 43.5 (SD 19.8) years | 2031 (66.3%) males | Not reported | Not reported | Not reported |
| Inagaki (2018) | 927/38 cervical spine injuries  The sample size was estimated based on 95% sensitivity with a 95% CI ± 5% | Median 59 (IQR 36-75) years | 587 (63.3%) males | - Ground-level fall: 379 (40.9%)  - Fall downstairs: 173(18.7%)  - Motor vehicle Collision: 93 (10.0%)  - Bicycle collision: 83 (9.0%)  - Motorcycle collision: 80 (8.6%)  - Assault: 42 (4.5%)  - Struck as pedestrian 33 (3.5%)  - Fall from ≥ 3 meter height: 11 (1.2%)  - Hanging: 2 (0.2%)  - Fall onto head: 1 (0.1%)  - Other: 29 (3.1%) | Not reported | Not reported |
| Khera (2022) | 1601/202 fractures | Mean 73.9 (range 65.4 to 96.8) years | All females | Not reported | Not reported | Not reported |
| Leonard (2011) | Cases: 540 cervical spine injuries  Random controls: 1060  Mechanism of injury controls: 1012  Emergency medical services controls: 702 | Cases:  - 0 to 2: 27  - 2 to 8: 140  - 8 to 16: 373  Random controls:  - 0 to 2: 116  - 2 to 8: 318  - 8 to 16: 626  Mechanism of injury controls:  - 0 to 2: 41  - 2 to 8: 264  - 8 to 16: 707  Emergency medical services controls:  - 0 to 2: 34  - 2 to 8: 173  - 8 to 16: 495 | Cases: 344 males (64%)  Random controls: 634 males (60%)  Mechanism of injury controls: 620 males (61%)  Emergency medical services controls: 414 males (59%) | Cases:  - Occupant of an automobile involved in an MVC: 151  - Nonautomobile MVC (includes children hit by cars and crashes  Involving motorcycles/all-terrain vehicles): 73  - Falls (includes falls from bikes and during sports; and diving): 193  - Other (includes other types of sport injuries and injuries involving  animals): 123  Random controls:  - Occupant of an automobile involved in an MVC: 259  - Nonautomobile MVC (includes children hit by cars and crashes  Involving motorcycles/all-terrain vehicles): 218  - Falls (includes falls from bikes and during sports; and diving): 386  - Other (includes other types of sport injuries and injuries involving  animals): 197  Mechanism of injury controls:  - Occupant of an automobile involved in an MVC: 276  - Nonautomobile MVC (includes children hit by cars and crashes  Involving motorcycles/all-terrain vehicles): 129  - Falls (includes falls from bikes and during sports; and diving): 368  - Other (includes other types of sport injuries and injuries involving  animals): 239  Emergency medical services controls:  - Occupant of an automobile involved in an MVC: 204  - Nonautomobile MVC (includes children hit by cars and crashes  Involving motorcycles/all-terrain vehicles): 185  - Falls (includes falls from bikes and during sports; and diving): 198  - Other (includes other types of sport injuries and injuries involving  animals): 115 | Not reported | Not reported |
| Roux (2007) | 397/not clear the number of patients with a fracture | Mean 74.3 (SD 5.5) years | All females | Not reported | Mean 62.5 on the VAS (SD 13.7) | Mean 61.2 months (SD 97.0) |
| Singh (2011) | 773 (261 fractures and 512 non-fratures) | Mean 41 years | 540 males (70%) | Cases:  - Motor vehicle accident: 89 (34.1%)  - Motorbike accident: 51 (19.5%)  - Struck ad pedestrian: 22 (8.4%)  - Falls: 72 (27.6%)  - Assault: 1 (0.4%)  - Cyclist: 7 (2.7%)  - Other: 19 (7.3%)  Controls:  - Motor vehicle accident: 140 (27.3%)  - Motorbike accident: 43 (8.4%)  - Struck ad pedestrian: 48 (9.4%)  - Falls: 138 (27.0%)  - Assault: 59 (11.5%)  - Cyclist: 16 (3.1%)  - Other: 68 (13.3%) | Cases:  153 (58.6%) had thoracic back pain  Controls:  50 (9.8%) had thoracic back pain | Not reported |
| Stiell (2001) | 8924/515 cervical spine injuries  Samples size estimated based on 100% sensitivity (95% CI 97%-100%) | Mean 36.7 (SD 16) years | 4600 males (51.5%) | - Motor vehicle collision: 5975 (67.0 %)  - Fall: 1277 (14.3%)  - Pedestrian struck: 298 (3.3%)  - Assault: 293 (3.3%)  - Head struck/hit by object: 291 (3.3%)  - Sports: 256 (2.9%)  - Bicycle: 221 (2.5%)  - Axial load: 192 (2.2%)  - Motorcycle: 66 (0.7%)  - Motorized recreational vehicle: 47 (0.5%)  - Other: 8 (0.1%) | Not reported | Not reported |
| Stiell (2003) | 8283/169 cervical spine injuries  Sample size estimation mentioned but method not reported | Mean 37.6 (SD 16) years | 4328 males (52.3%) | - Motor vehicle collision: 5564 (67.2%)  - Motorcycle collision: 78 (0.9%)  - Collision involving other motorized vehicles: 53 (0.6%)  Pedestrian struck and thrown: 107 (1.3%)  - Pedestrian struck: 158 (1.9%)  Bicycle struck: 96 (1.2%)  - Bicycle collision: 61 (0.7%)  - Other bicycle accident: 105 (1.3%)  - Fall from elevation >10 ft (3 m) or down >15 stairs: 183 (2.2%)  Fall from elevation of 3 to 10 ft (1 to 3 m) or down 5 to 15 stairs:  350 (4.2%)  - Fall from elevation <3 ft (1 m) or down <5 stairs: 641 (7.7%)  - Assault with a blunt object: 73 (0.9%)  - Assault with fist or feet: 199 (2.4%)  - Diving: 25 (0.3%)  - Fall onto head (axial load): 32 (0.4%)  - Contact sports (axial load): 88 (1.1%)  - Heavy object onto head (axial load): 74 (0.9%)  - Other sports: 166 (2.0%)  - Head struck by other object: 106 (1.3%)  - Hit head on an object: 101 (1.2%)  - Other: 23 (0.3%) | Not reported | Not reported |
| Stiell (2010) | 3411/41 cervical spine injuries | Mean 41 (SD 18) years | 1687 males (36.4%) | - Motor vehicle collision: 2288 (63.0%)  - Pedestrian struck: 98 (2.7%)  - Bicycle collision: 133 (3.7%)  - Fall: 679 (18.7%) Assault: 58 (1.6%)  - Axial load (e.g., diving, fall, sports): 60 (1.7%)  - Hit head on an object: 170 (4.7%)  - Other: 60 (1.7%) | Not reported | Not reported |
| Vaillancourt (2009) | 1947/12 cervical spine injuries for paramedics  1629/12 cervical spine injuries for study investigators | Median 39 years (IQR 26-52, range 16-103) years | 1403 males (49.3%) | - Motor vehicle crash: 1,218 (62.5%)  - Motorcycle crash: 41 (2.1%)  - Crash involving other motorized vehicles: 21 (1.1%)  - Bicycle struck: 36 (1.8%)  - Bicycle crash: 25 (1.3%)  - Other bicycle accidents: 29 (1.5%)  - Pedestrian struck: 44 (2.3%)  - Pedestrian struck and thrown: 30 (1.5%)  - Fall from elevation 3 ft (1 m) or down 5 stairs: 209 (10.7%)  - Fall from elevation of 3–10 ft (1–3 m) or down 5–15 stairs: 108 (5.5%)  - Fall from elevation 10 ft (3 m) or down 15 stairs: 70 (3.6%)  - Fall onto head (axial load): 1 (0.1%)  - Heavy object onto head (axial load): 9 (0.5%)  - Contact sport (axial load): 16 (0.8%)  - Diving: 2 (0.1%)  - Other sport: 21 (1.1%)  - Assault with fist or feet: 33 (1.7%)  - Assault with a blunt object: 14 (0.7%)  - Head struck by other object: 11 (0.6%)  - Hit head on an object: 5 (0.3%)  Other: 6 (0.3%) | Not reported | Not reported |
| Vaillancourt (2023) | 4021/11 cervical spine injuries for paramedics  3842/11 cervical spine injuries for study investigators | Mean 42.9 years (range 16-99) years | 1881 males (46.9%) | - Motor vehicle collision: 2,221 (55.1%)  - Motorcycle: 62 (1.5%)  - Other motorized vehicle: 24 (0.6%)  - Fall from sitting: 41 (1.0%)  - Fall from standing: 592 (14.7%)  - Fall from elevation < 3 feet/5 stairs: 124 (3.1%)  - Fall from elevation 3-10 feet/5 to 15 stairs: 163 (4.0%)  - Fall from elevation >10 feet/15 stairs: 45 (1.1%)  - Assault fist or feet: 148 (3.7%)  - Assault blunt object: 37 (0.9%)  - Struck: 105 (2.6%)  - Pedestrian struck and thrown: 29 (0.7%)  - Hit head on an object: 224 (5.6%)  - Head struck by other object: 30 (0.7%)  - Fall onto head (axial load): 10 (0.2%)  - Heavy object onto the head (axial load): 8 (0.2%)  - Other bicycle: 84 (2.1%)  - Bicycle struck: 47 (1.2%)  - Bicycle collision: 36 (0.9%)  - Other sports: 60 (1.5%)  - Contact sports (axial load): 2 (0.0%)  - Diving: 2 (0.0%)  - Other: 185 (4.6%) | Not reported | Not reported |
