# Supplementary material for: Diagnostic prediction models for spinal fractures in individuals with spinal pain or trauma: a systematic review and meta-analysis
Source: eClinicalMedicine. 2025 Aug 26;88:103456. doi: 10.1016/j.eclinm.2025.103456 (PMC12572814; doi:10.1016/j.eclinm.2025.103456)
Supplement: Supplementary Material 6 [file mmc6.docx]

| **First author (year)** | **Modeling method** | **Handling of predictors** | **Model assumptions** | **Method for selection of predictors for inclusion in multivariable modelling** | **Method for selection of predictors during multivariable modelling** | **Internal validation** |
| --- | --- | --- | --- | --- | --- | --- |
| Athinartrattanapong (2021) | Logistic regression | Categorization of continuous variables | Not reported | Univariate association (threshold for the p - values not specified), high discriminative performance (AUC), and clinical relevance | Not performed | Not performed |
| Bandiera (2003) | Recursive partitioning | Not reported | Not reported | Not applicable | Not applicable | Not performed |
| Bub (2005) | Recursive partitioning | Independent predictors were combined into composite predictors | Not reported | Not reported | Various models were constructed and evaluated by their ROC curves and clinical relevance | 1000 bootstrap samples |
| Caltili (2017) | Not applicable | Not applicable | Not applicable | Not applicable | Not applicable | Not applicable |
| Clark (2016) | Logistic regression | No reported information, although some potentially continuous variables (e.g. duration of pain) were presented as dichotomous | Not reported | Unviariate association (p – value < 0.1) | Backward selection based on the p – value and the pseudo R – squared.  All excluded variables were reintroduced into the final model to check that the decision to eliminate them was correct | Not performed |
| Coffrey (2015) | Not applicable | Not applicable | Not applicable | Not applicable | Not applicable | Not applicable |
| Cook (2013) | Logistic regression | Continuous and categorical variables were dichotomized | Not reported | Univariate association (LR+ > 1.5 or LR- < 0.5) | Backward stepwise selection (p – value > 0.15 to exit the model and p – value < 0.10 to enter it) | Not performed |
| Duane (2011) | V: Not applicable  D: Logistic regression | V: Not applicable  D: continuous variables dichotomized | V: Not applicable  D: Not reported | V: Not applicable  D: Based on variables from the Canadian C-spine and univariate association | V: Not applicable  D: Stepwise selection | V: Not applicable  D: Not performed |
| Duane (2013) | V: Not applicable  D: Logistic regression | V: Not applicable  D: Continuous variables dichotomized | V: Not applicable  D: Not reported | V: Not applicable  D: Based on predictors included in the NEXUS and Canadian C-spine rules, and on univariate association (cut–off of the p - value not reported) | V: Not applicable  D: Stepwise selection | V: Not applicable  D: Not performed |
| Ehrlich (2009) | Not applicable | Not applicable | Not applicable | Not applicable | Not applicable | Not applicable |
| Engelbart (2021) | Logistic regression | Categorization of continuous variables | Not reported | Variables representing demographic characteristics, NEXUS criteria  and distracting injuries | Models were fit for all subset combinations with a maximum model order of four, using the (smallest) AIC to choose the best model | Split–sample approach (validation set n = 1605) |
| Enthoven (2016) | Logistic regression | Categorization of continuous variables | Multicollinearity was checked (  (Pearson’s correlation > 0.6) | Univariate association (p – value < 0.05) of red flags and other  determinants that were considered  important for diagnosing vertebral fractures | Backward  selection (cutoff for removal p – value > 0.1) | Not performed |
| Ghelichkhani (2021) | Not applicable | Not reported | Not applicable | Not applicable | Not applicable | Not applicable |
| Henschke (2009) | Not reported | Dichotomization of continuous variables | Not reported | Based on previous literature, consultation with experts in  the field, and univariate association (p – value < 0.1) | Not performed | Not performed |
| Hercz (2019) | Recursive partitioning | Dichotomization of continuous and categorical variables | Not reported | Based on a review of the literature | Not performed | Not performed |
| Ikemoto (2022) | Logistic regression | Categorical variables (3 categories) were managed as continuous in the regression model | Not reported | Based on a review of the literature | Stepwise selection | Not performed |
| Inaba (2015) | Logistic regression | Dichotomization of continuous and categorical variables | Not reported | Based on a literature review and univariate association | Not performed | Not performed |
| Inagaki (2018) | V: Not applicable  D: Recursive partitioning | V: In rule already dichotomized/categorized  D: Dichotomization of continuous variables | V: Not reported  D: Not reported | V: Not applicable  D: Based on clinical feasibility | V: Not applicable  D: Not performed | V: Not applicable  D: Not performed |
| Khera (2022) | Logistic regression | Continuous predictor variables were investigated as to whether the model could be improved by their prior transformation using fractional polynomials up to the second degree | Not reported | Based on previous literature and univariate association (p – value < 0.1) | Backward selection (removal of variables with a p – value > 0.1) on various subsets of the collected variables. The reduced subsets of predictor variables were then combined and analysed with a similar backward stepwise approach.  Age was forced to be in the model | 500 bootstrap samples |
| Leonard (2011) | Logistic regression | Dichotomization of continuous variables | Not reported | Based on previous literature and biological plausibility | Forward selection considering all potential variables, adding the largest X^2^ until no remaining variable scored a p – value < 0.5 | 1000 bootstrap samples |
| Roux (2007) | Logistic regression | Categorization of continuous variables | Not reported | Univariate association (p – value < 0.2) | Stepwise selection (p – value < 0.1) | Not reported |
| Singh (2011) | Logistic regression | Dichotomization of continuous variables | Not reported | Univariate association (Pearson’s chi-square, Fisher’s exact and Mann-Whitney U test) using a p – value < 0.05 | Stepwise selection, with the best model being based on the p-value, AUC, the Hosmer test, and the percentage of correctly classified cases | Not performed |
| Stiell (2001) | Model 1: Recursive partitioning  Model 2: Logistic regression | Variables were dichotomized or categorized | Not reported | Univariate association (p - value < 0.05) and high interobserver agreement in the assessment (k coefficient > 0.6) | Model 2: Stepwise selection (entry criteria: p - value < 0.05, removal criteria: p - value < 0.10) | Cross-validation with jackknife nonparametric estimation of bias |
| Stiell (2003) | Not applicable | Not applicable | Not reported | Not applicable | Not applicable | Not applicable |
| Stiell (2010) | Not applicable | Not applicable | Not reported | Not applicable | Not applicable | Not applicable |
| Vaillancourt (2009) | Not applicable | In rule already dichotomized/categorized | Not reported | Not applicable | Not applicable | Not applicable |
| Vaillancourt (2023) | Not applicable | In rule already dichotomized/categorized | Not reported | Not applicable | Not applicable | Not applicable |
