# Supplementary material for: Diagnostic prediction models for spinal fractures in individuals with spinal pain or trauma: a systematic review and meta-analysis
Source: eClinicalMedicine. 2025 Aug 26;88:103456. doi: 10.1016/j.eclinm.2025.103456 (PMC12572814; doi:10.1016/j.eclinm.2025.103456)
Supplement: Supplementary Material 7 [file mmc7.docx]

| **Author (year)** | **Model presentation (sufficient/insufficient)** | **Candidate predictors to be included in the multivariable modeling** | **Final model** |
| --- | --- | --- | --- |
| Athinartrattanapong (2021) | Insufficient (missing intercept), but sufficient to be used in clinical practice as probability categories based on a score | Not reported | Associated chest or facial injury (OR 2.72, 95% CI 1.11 to 6.66) + High-risk mechanism (OR 2.94, 95% CI 1.10 to 7.87) + Paraparesis (OR 5.38, 95% CI 1.33 to 21.70) + Paresthesia (OR 1.66, 95% CI 0.30 to 9.22) + Limited neck ROM (OR 3.77, 95% CI 1.39 to 10.18) |
| Bandiera (2003) | Sufficient | Not applicable | Canadian C-spine rule |
| Bub (2005) | Sufficient to be applied, but insufficient to be replicated | Not reported | - Focal neurological deficit (if yes: 24.2% with 95% CI 5.7% to 100% of having a fracture; if no: next step)  - Severe head injury (intracranial hemorrhage, skull fracture, and/or unconsciousness at examination) (if yes: 7.9% with 95% CI 3.0% to 19.6% of having a fracture; if no: next step)  - High – energy mechanism (high-speed motor vehicle crash, a fall from 3 meters, a car striking a pedestrian, or airplane accident) (if yes: 3.4% with 95% CI 1.2% to 10.1% of having a fracture; if no: next step)  - Moderate energy mechanism (low-speed motor vehicle crash, a fall from less than 3 meters, or a skiing accident) (if yes: 1.4% with 95% CI 0.3% to 1.6% of having a fracture; if no: next step)  - Low – energy mechanism (fall from standing or sitting position) (if yes: 0.4% with 95% CI 0.1% to 1.3% of having a fracture) |
| Caltili (2017) | Rule mentioned, not reported | Not applicable | Canadian C-spine rule |
| Clark (2016) | Insufficient (missing intercept) | Not reported | Age (OR 1.09, 95% CI 1.03 to 1.16) + History of previous fracture (OR 2.91, 95% CI 1.13 to 7.52) + Short duration of back pain (days or weeks) (OR 5.98, 95% CI 2.31 to 15.50) + Pain described as crushing (OR 4.35, 95% CI 1.01 to 18.74) + Pain improving on lying down (OR 3.34, 95% CI 1.05 to 10.69) + Pain not spreading down the legs (OR 0.21, 95% CI 0.07 to 0.61) |
| Coffrey (2015) | Sufficient | Not applicable | Canadian C-spine rule. In 202 cases, doctors did not evaluate range of motion as required by the rule |
| Cook (2013) | Insufficient (missing intercept and coefficients) to be replicated, but sufficient to be used in clinical practice | Marital status (single), duration of symptoms < 6 weeks, condition involved an emergency room visit, non-full-time work status, age ≤ 33 years, no prior history of physical therapy | Marital status (single) + Duration of symptoms < 6 weeks + Condition involved an emergency room visit + Non-full-time work status + Age ≤ 33 years + No prior history of physical therapy |
| Duane (2011) | V: Sufficient  D: Insufficient (missing intercept) | V: Not applicable  D: All variables of the Canadian C-spine rule except for cervical rotation | V: Canadian C-spine rule without range of motion in cervical rotation  D: table 3 |
| Duane (2013) | V: Sufficient  D: Insufficient (missing intercept) | V: Not applicable  D: All variables of the NEXUS and Canadian C-spine rule except for cervical rotation | V: Canadian C-spine rule without range of motion in cervical rotation  D: Midline tenderness (OR 3.52, 95% CI 2.73 to 4.53) + GCS score <15 (OR 2.60, 95% CI 2.00 to 3.39) + Aged ≥ 65 yr (OR 2.07, 95% CI 1.48 to 2.90) + Paresthesia (OR 2.39, 95% CI 1.58 to 3.62) + Rollover MVC (OR 1.70, 95% CI 1.30 to 2.21) + Patient ejected (OR 1.90, 95% CI 1.35 to 2.69) + Never in sitting position in ED (OR 5.49, 95% CI 3.18 to 9.47) |
| Ehrlich (2009) | Sufficient | Not applicable | Canadian C-spine rule |
| Engelbart (2021) | Insufficient (missing intercept) | Not reported | Model 1: Signs of trauma (OR 2.348, 95% CI 1.553 to 3.549) + Focal neurological deficit (OR 2.947, 95% CI 1.326 to 6.551) + Midline spinal tenderness (OR 22.961, 95% CI 15.178 to 34.737)  Model 2 (subsample of patients where midline tenderness was noted as no or unknown): Signs of trauma (OR 3.024, 95% CI 1.898 to 4.815) + Focal neurological deficit (OR 2.601, 95% CI 1.340 to 5.049) |
| Enthoven (2016) | Insufficient (missing intercept) | Age ≥ 75 years, prolonged corticosteroid use, trauma, osteoporosis, severe disability (RMDS > 17), back pain intensity score of ≥ 7, thoracic back pain | Age ≥ 75 years (OR 3.5, 95% CI 1.5 to 8.6) + Trauma (OR 7.8, 95% CI 2.7, 22.5) + Osteoporosis (OR 2.5 95% CI 1.0 to 6.2) + Back pain intensity score of ≥ 7 (OR 3.1 95% CI 1.4 to 7.2) + Thoracic back pain (OR 2.1 95% CI 0.9 to 4.9). |
| Ghelichkhani (2021) | Rule mentioned, not reported | Not applicable | Canadian C-spine rule |
| Henschke (2009) | Insufficient (missing intercept and coefficients) to be replicated, but sufficient to be used in clinical practice | Age > 70 years, significant trauma (major in young, minor in elderly), prolonged use of corticosteroids, sensory level (altered sensation from trunk down), clinician diagnosis of fracture, and gender | Gender (female) + Age > 70 years + Significant trauma (major in young, minor in elderly) + Prolonged use of corticosteroids |
| Hercz (2019) | Insufficient (missing intercept and coefficients) to be replicated, but sufficient to be used in clinical practice | Not reported | Midline tenderness + Focal neurological deficit + Mechanism of injury (any fall above ground level, high risk MVC, unenclosed MVC including pedestrian and bicyclist hit by car, and assault with any weapon) + Age ≥ 65 |
| Ikemoto (2022) | Insufficient (missing intercept) to be replicated, but sufficient to be used in clinical practice | Age, sex, pain duration, pain severity, difficulty in getting up (score 0 to 2), and difficulty in rolling over (score 0 to 2) | Difficulty in rolling over (OR 8.56, 95% CI 2.33 to 31.46) + Difficulty in getting up (OR 5.03, 95% CI 1.24 to 20.39) + Age (OR 1.09, 95% CI 1.00 to 1.20) |
| Inaba (2015) | Model insufficient (missing intercept and coefficients)  Rule reported | Pain, midline, tenderness to palpation, deformity, neurological deficit, various injury mechanisms, age. | Model: Age ≥ 60 + high-risk mechanism + positive physical examination finding  Rule: Alert and evaluable, or Positive physical exam (pain, tenderness to palpation, deformity, neurologic deficit) or High-risk mechanism (fall, crush injury, MVC with rollover/ejection, unenclosed vehicle crash, automobile versus pedestrian) or Age ≥ 60 years |
| Inagaki (2018) | V: Sufficient (rule reported)  D: Sufficient (rule reported) | V: Not applicable  D: Not reported | V: GCS score < 14, posterior cervical tenderness or neurological deficit, and GCS  score 14–15, age ≥60 years who have fallen downstairs, or age <60 who have been injured in a motorcycle collision or fallen from height  D: GCS score <14, cervical tenderness, neurological deficit, falling downstairs, motorcycle collision or fall from height |
| Khera (2022) | Sufficient | Not reported | Age (OR 0.98, 95% CI 0.94 to 1.01) + Weight (OR 0.98, 95% CI 0.96 to 0.99) + Wall to tragus (OR 1.07, 95% CI 1.01 to 1.13) + Reported height loss (OR 1.17, 95% CI 1.10 to 1.25) + Pain described as sharp (OR 0.63, 95% CI 0.40 to 0.99) + Pain described as like tootache (OR 0.49, 95% CI 0.27 to 0.91) + Agreement with “If I’m working in the kitchen like chopping vegetables or washing my back pain get worse and worse to reach a peak – then I have to sit down immediately” (OR 1.97, 95% CI 1.30 to 3.00) + Pain in the thoracic area of the Margolis diagram (OR 1.66, 95% CI 1.11 to 2.49) + Pain the low back/buttock are of the Margolis diagram (OR 0.64, 95% CI 0.44 to 0.94) + Pain increased by walking (OR 0.55, 95% CI 0.37 to 0.84) + Pain affected by sitting on straight backed chairs (OR 1.78, 95% CI 1.16 to 2.74) + Pain affected by sitting on soft chairs (OR 0.48, 95% CI 0.32 to 0.71) + Pain increased by reclining (OR 1.93, 95% CI 1.24 to 3.02) + Fractures after age 50, excluding hands, feet, head and excluding high trauma (OR 3.33, 95% CI 2.30 to 4.82) + Steroids > 3 months (OR 1.37, 95% CI 0.81 to 2.32) + Constant (coefficient of – 1.93, standard error of 1.46) |
| Leonard (2011) | Insufficient (missing intercept) as a model, but sufficient as rule | Altered mental status, loss of consciousness, non-ambulatory, focal neurologic findings, complaint of neck pain, posterior midline neck tenderness, any neck tenderness, torticollis, substantial injury (extremity, face, head, torso), predisposing condition, high-risk mechanism (diving, fall, hanging, hit by car, MVC, other MV, axial load any region head, on top of head), clothes lining | Random controls model: Altered mental status (OR 3.0, 95% CI 2.1 to 4.3) + Focal neurologic deficit (OR 8.3, 95% CI 5.6 to 12.2) + Complaint of neck pain (OR 3.2, 95% CI 2.3 to 4.4) + Torticollis (OR 1.8, 95% CI 1.1 to 2.9) + Predisposing condition (OR 15.0, 95% CI 2.9 to 78.0) + Substantial injury to the torso (OR 1.9, 95% CI 1.1 to 3.4) + Diving (OR 73.0, 95% CI (9.6 to 555.6) + High-risk motor vehicle crash (OR 2.5, 95% CI 1.8 to 3.6)  Two models were also fitted to the other two control groups. The variables common to all models were altered mental status, focal neurologic deficit, complaint of neck  pain, substantial injury to the torso, high-risk motor vehicle crash, and diving |
| Roux (2007) | Insufficient (missing intercept) | Not reported | Age (per 1 year) (OR 1.076, 95% CI 1.031 to 1.123) + Age (per 5 years) (OR 1.444, 95% CI 1.165 to 1.788) + Score ≥ 65 non the VAS for pain (OR 1.846, 95% CI 1.171 to 2.911) + Height loss between 3 and 6 cm (OR 1.939, 95% CI 1.081 to 3.479) + Height loss ≥ 6 cm (OR 3.229, 95% CI 1.688 to 6.176) + Pain in the thoracic area (OR 1.624, 95% CI 1.031 to 2.560) + Sudden occurrence of pain (OR 3.370, 95% CI 2.093 to 5.424) + History of low-trauma peripheral fracture (pelvis, forearm, rib, hip) (OR 1.615, 95% CI 0.947 to 2.755) |
| Singh (2011) | Insufficient (missing intercept) | Motor vehicle accident speed (high, medium, low), restraint (restrained or unrestrained), designation (driver, front-seat passenger, rear-seat passenger, other), MBA speed (high, medium, low), helmet (helmeted, not helmeted), struck as pedestrian speed (high, medium, low), fall height (high, medium, low), assault, cyclist, other, thoracic back pain (yes/no), thoracic spine tenderness (yes/no), intoxication (yes/no), Glasgow Coma Score < 15, intubated prior to emergency department (yes/no), neurological symptoms (yes/no), neurological signs (yes/no), step deformity (yes/no). | Fall from height ≥ 2 (OR 7.0, 95% CI 13.0 to 16.3) + Presence of back pain (OR 13.6, 95% CI 5.8 to 31.4) + Intoxication (OR 0.5, 95% CI 0.3 to 0.8) |
| Stiell (2001) | Model 1: Sufficient  Model 2: Sufficient (rule presented) | Not reported | Model 1: Logistic regression model: - 3.63 (intercept) + Dangerous mechanism (OR 5.3, 95% CI 3.7 to 7.3) + Age ≥ 65 (OR 3.7, 95% CI 2.4 to 5.6) + Paraesthesias in extremities (OR 2.2, 95% CI 1.4 to 3.3) + Ambulatory at any time after injury (OR 1.9, 95% CI 0.7 to 1.5) + Sitting position in ED (OR 0.61, 95% CI 0.3 to 1.2) + Delayed onset of neck pain (OR 0.4, 95% CI 0.3 to 0..7) + Absence of midline neck tenderness (OR 0.5, 95% CI 0.3 to 0.8) + Able to rotate neck 45 left and right (OR 0.04, 95% CI 0.01 to 0.3) + Simple rear-end MVC (OR 0.08, 95% CI 0.03 to 0.2).  Model 2: Canadian C-spine rule |
| Stiell (2003) | Rule reported | Not applicable | Canadian C-spine rule |
| Stiell (2010) | Rule reported | Not applicable | Canadian C-spine rule |
| Vaillancourt (2009) | Rule reported | Not applicable | Canadian C-spine rule, with the removal of the low-risk criteria “delayed onset of neck pain” |
| Vaillancourt (2023) | Rule reported | Not applicable | Canadian C-spine rule, with the removal of the low-risk criteria “delayed onset of neck pain” |
