# Supplementary material for: Diagnostic prediction models for spinal fractures in individuals with spinal pain or trauma: a systematic review and meta-analysis
Source: eClinicalMedicine. 2025 Aug 26;88:103456. doi: 10.1016/j.eclinm.2025.103456 (PMC12572814; doi:10.1016/j.eclinm.2025.103456)
Supplement: Supplementary Material 8 [file mmc8.docx]

| **Author (year)** | **Calibration measures** | **Discrimination measures** | **Classification measures** |
| --- | --- | --- | --- |
| Athinartrattanapong (2021) | Plot of predicted risk score versus % observed risk (good calibration) | AUC 0.83 (95% CI 0.74 to 0.91) | Low risk (score of 0):  LR+ 0.18 (95% CI 0.05 to 0.56)  Moderate risk (score between 1 and 5):  LR+ 1.46 (95% CI 1.09 to 1.96)  High risk (score between 6 and 11):  LR+ 7.16 (95% CI 2.82 to 18.19) |
| Bandiera (2003) | Not reported | AUC 0.91 (95% CI 0.89 to 0.92) | - Sensitivity 100% (95% CI 94% to 100%)  - Specificity 44.0% (95% CI 43% to 45%) |
| Bub (2005) | Not reported | AUC 0.82 | Not reported |
| Caltili (2017) | Not reported | Not reported | - Sensitivity 99.7%  - Specificity 17.9%  - PPV 16.3%  - NPV 99.7% |
| Clark (2016) | Not reported | AUC 0.85 (95% CI 0.79 to 0.92) | With a cut-off of 0.39:  - Sensitivity 77%  - Specificity 78% |
| Coffrey (2015) | Not reported | Not reported | - Sensitivity 100% (95% CI 56% to 100%)  - Specificity 43% (95% CI 39% to 45%) |
| Cook (2013) | Not reported | Not performed | Presence of 1 of 6 predictors:  - Sensitivity 100% (95% CI 100% to 100%)  - Specificity 15% (95% CI 9% to 21%)  - LR+ 1.17 (95% CI 1.1 to 1.3)  Presence of 2 of 6 predictors:  - Sensitivity 100% (95% CI 100% to 100%)  - Specificity 50% (95% CI 42% to 58%)  - LR+ 2.0 (95% CI 1.7 to 2.3)  Presence of 3 of 6 predictors:  - Sensitivity 91% (95% CI 74% to 100%)  - Specificity 84% (95% CI 78% to 90%)  - LR+ 5.7 (95% CI 3.8 to 8.6)  - LR- 0.11 (95% CI 0.02 to 0.70)  Presence of 4 of 6 predictors:  - Sensitivity 64% (95% CI 35% to 92%)  - Specificity 98% (95% CI 96% to 100%)  - LR+ 32 (95% CI 9.6 to 107)  - LR- 0.37 (95% CI 0.2 to 0.8)  Presence of 5 of 6 predictors:  - Sensitivity 18% (95% CI 0% to 41%)  - Specificity 100% (95% CI 100% to 100%)  - LR- 0.82 (95% CI 0.6 to 1.1) |
| Duane (2011) | Not reported | Developed model:  AUC 0.77 | Validation of the Canadian C-spine:  - Sensitivity 100%  - Specificity 0.60%  - PPV 6.03%  - NPV 100% |
| Duane (2013) | Not reported | Developed model:  AUC 0.75 | Developed model:  - Sensitivity 99.07%  - Specificity 11.57%  - PPV 6.95%  - NPV 99.47%  Validation of the Canadian C-spine:  - Sensitivity 100%  - Specificity 0.62%  - PPV 6.29%  - NPV 100% |
| Ehrlich (2009) | Not reported | Not reported | - Sensitivity 86%  - Specificity 94% |
| Engelbart (2021) | Not reported | Model 1 (using a probability threshold of 0.3):  AUC (validation set) 0.82  AUC (training set) 0.83  Model 2 (using a probability threshold of 0.1):  AUC (validation set) 0.65  AUC (training set) 0.66 | Model 1 (using a probability threshold of 0.3):  - Sensitivity 61.1% (95% CI 53.2% to 68.9%)  - Specificity 93.7% (95% CI 92.3% to 95.0%)  - NPV 95.1% (95% CI 93.9% to 96.3%)  Model 2 (using a probability threshold of 0.1):  - Sensitivity 8.1% (95% CI 2.4% to 13.9%)  - Specificity 97.4% (95% CI 96.5% to 98.2%)  - NPV 94.3% (95% CI 93.1% to 95.5%)  Model 2 (applied to all patients independently from the presence of midline tenderness):  - Sensitivity 86.6%  - Specificity 54.5%  - NPV 97.1%  - PPV 18.9% |
| Enthoven (2016) | Not reported | AUC 0.78 (range 0.69 to 0.87)  Model’s Nagelkerke R^2^: 20.3% (overall measure of performance) | ≥ 1 positive feature:  - Sensitivity 88% (95% CI 77% to 995)  - Specificity 42% (95% CI 38% to 46%)  - LR+ 1.5 (95% CI 1.3 to 1.8)  - LR- 0.3 (95% CI 0.1 to 0.7)  - PPV 7% (95% CI 5% to 10%)  - NPV 99% (95% CI 97% to 100%)  ≥ 2 positive features:  - Sensitivity 70% (95% CI 54% to 85%)  - Specificity 81% (95% CI 78% to 84%)  - LR+ 3.6 (95% CI 2.8 to 4.8)  - LR- 0.4 (95% CI 0.2 to 0.6)  - PPV 16% (95% CI 10% to 22%)  - NPV 98% (95% CI 97% to 99%)  ≥ 3 positive features:  - Sensitivity 30% (95% CI 15% to 46%)  - Specificity 95% (95% CI 93% to 97%)  - LR+ 5.8 (95% CI 3.2 to 10.8)  - LR- 0.7 (95% CI: 0.6 to 0.9)  - PPV 23% (95% CI 11% to 36%)  - NPV 96% (95% CI 95% to 98%) |
| Ghelichkhani (2021) | Not reported | AUC 0.78 (95% CI 0.74 to 0.83) | - Sensitivity: 100% (95% CI 91.3% to 100%)  - Specificity: 8.3% (95% CI 6.4% to 10.9%)  - LR+: 1.1 (95% CI 1.1 to 1.1)  - LR-: 0 (not applicable)  - PPV 9.8% (95% CI 7.6% to 12.5%)  - NPV 100% (95% CI 91.3% to 100%) |
| Henschke (2009) | Not reported | AUC 0.83 (95% CI: 0.65 to 1; p – value 0.001) | One positive feature:  - Sensitivity 88%  - Specificity 50%  - LR+ 1.8 (95% CI: 1.1 to 2.0)  Two positive features:  - Sensitivity 63%  - Specificity 96%  - LR+ 15.5 (95% CI 7.2 to 24.6)  Three positive features:  - Sensitivity 38%  - Specificity 100%  - LR+ 218.3 (95% CI 45.6 to 953.8) |
| Hercz (2019) | Not reported | Not reported | - Sensitivity 100% (95% CI 91.2% to 100%)  - Specificity: 53.1% (95% CI 50.5% to 55.7%)  - NPV 100% (95% CI 99.3% to 100%) |
| Ikemoto (2022) | Not reported | AUC 0.88 (p – value < 0.01) | Difficulty in getting up and rolling over sum score of 2:  - Sensitivity 97.5%  - Specificity 32.5%  Difficulty in getting up and rolling over sum score of 3:  - Sensitivity 87.5%  - Specificity 75.0%  Difficulty in getting up and rolling over sum score of 4:  - Sensitivity 65.0%  - Specificity 95.0% |
| Inaba (2015) | Not reported | Model:  AUC 0.81 (95% CI 0.78 to 0.83) | Rule:  - Sensitivity 98.9%  - Specificity 29.0%  - PPV 11.6%  - NPV 99.6% |
| Inagaki (2018) | Not reported | Not reported | Development phase:  - Sensitivity: 100% (95% CI 90.8% to 100%)  - Specificity 51.9 % (95% CI 48.6% to 55.2%)  - NPV 100%  Validation phase:  - Sensitivity 92.1% (95% CI 79.2% to 97.3%)  - Specificity 58.6 % (95% CI 55.4% to 61.9%)  - NPV 99.4% |
| Khera (2022) | Calibration slope 1.0 | AUC 0.80 (95% CI 0.76 to 0.84)  Optimized-adjusted AUC 0.78 | Using a cut-off of the linear predictor of – 2.0:  - Sensitivity 72.4%  - Specificity 72.9%  - PPV 27.1%  - NPV 95.0% |
| Leonard (2011) | Not reported | Not reported | Presence of at least 1 variable of the random control model:  - Sensitivity 94% (95% CI 91% to 96%)  - Specificity 32% (95% CI 29% to 35%)  Addition to the random control model of positive findings from transferring hospital emergency department or emergency medical services:  - Sensitivity 98% (95% CI 96% to 99%)  - Specificity 26% (95% CI 23% to 29%)  Presence of at least 1 variable of the variables common to all models:  - Sensitivity 92% (95% CI 89% to 94%)  - Specificity 35% (95% CI 32% to 38%)  Addition of positive findings from transferring hospital emergency department or emergency medical services:  - Sensitivity 97% (95% CI 95% to 98%)  - Specificity 29% (95% CI 26% to 32%) |
| Roux (2007) | Not reported | AUC 0.77 | - PPV 70.9%  - NPV 68.6% |
| Singh (2011) | Not reported | AUC 0.88 | - Sensitivity 64%  - Specificity 93% |
| Stiell (2001) | Model (logistic regression):  Hosmer-Lemeshow test: p = 0.94 | Model 1 (logistic regression):  AUC 0.91 | Model 2 (Canadian C-spine rule):  - Sensitivity 100% (95% CI 98% to 100%)  - Specificity 42.5% (95% CI 40% to 44%)  - Bias corrected sensitivity 100%  - Bias corrected specificity 42.63% |
| Stiell (2003) | Not reported | Not reported | Results on 7438, excluding 845 indeterminate cases:  - Sensitivity 99.4% (95% CI 96% to 100%)  - Specificity 45.1% (95% CI 44% to 46%)  - NPV 100%  Results on all the included patients when the rule was assumed to be positive in all the indeterminate cases:  - Sensitivity 99.4% (95% CI 96% to 100%)  - Specificity 40.4% (95% CI 39% to 42%)  Results on all the included patients, when the rule was assumed to be negative in all the indeterminate cases:  - Sensitivity 95.3% (95% CI 91% to 97%)  - Specificity 50.7% (95% CI 50% to 52%) |
| Stiell (2010) | Not reported | Not reported | When applied by the nurse:  - Sensitivity 90.2% (95% CI 76% to 95%)  - Specificity 43.9% (95% CI 42% to 46%)  - NPV: 99.7%  When applied by the investigators:  - Sensitivity 100% (95% CI 91% to 100%)  - Specificity: 43.4% (95% CI 42.0% to 45.0%)  - NPV 100% |
| Vaillancourt (2009) | Not reported | Not reported | When applied by the paramedics:  - Sensitivity 100% (95% CI 74% to 100%)  - Specificity 37.7% (95% CI 36% to 40%)  - NPV 100% (95% CI 99% to 100%)  When applied by the investigators:  - Sensitivity 100% (95% CI 74% to 100%)  - Specificity 42.9% (95% CI 40% to 45%)  - NPV 100% (95% CI 99% to 100%)  All indeterminate cases assumed positive:  - Specificity 32.4% (95% CI 31% to 34%)  All indeterminate cases assumed negative:  - Specificity 46.6% (95% CI 45% to 49%) |
| Vaillancourt (2023) | Not reported | Not reported | When applied by the paramedics:  - Sensitivity 90.9% (95% CI 58.7% to 99.8%)  - Specificity 66.5% (95% CI 65.1% to 68.0%)  - LR+ 2.7 (95% CI 2.2 to 3.4)  - LR- 0.1 (95% CI 0.0 to 0.9)  When applied by the investigators:  - Sensitivity 90.9% (95% CI 58.7% to 99.8%)  - Specificity 68.2% (95% CI 66.7% to 69.7%)  - LR+ 2.9 (95% CI 2.4 to 3.5)  - LR- 0.1 (95% CI 0.0 to 0.9) |
