# Supplementary material for: Diagnostic prediction models for spinal fractures in individuals with spinal pain or trauma: a systematic review and meta-analysis
Source: eClinicalMedicine. 2025 Aug 26;88:103456. doi: 10.1016/j.eclinm.2025.103456 (PMC12572814; doi:10.1016/j.eclinm.2025.103456)
Supplement: Supplementary Material 9 [file mmc9.docx]

| **Author (year)** | **Total number of patients with missing data** | **Number of missing in the predictors** | **Number of missing in the outcome** | **Methods for handling missing data** |
| --- | --- | --- | --- | --- |
| Athinartrattanapong (2021) | Not reported | Not reported | Not reported | Not reported |
| Bandiera (2003) | Not reported | Not reported | Not reported | Not reported |
| Bub (2005) | 1 | 1 | 0 | Complete – case analysis |
| Caltili (2017) | 20 (unclear whether the missing data pertained to predictors, outcomes, or general patients information) | Not reported | Not reported | Not reported |
| Clark (2016) | Not reported | Not reported | Not reported | Complete – case analysis |
| Coffrey (2015) | 354 | 202 (one variable of the rule is missing) | 152 (unable to contact at the 14 days follow-up) | Complete – case analysis |
| Cook (2013) | 102 | Not reported | Not reported | Complete – case analysis |
| Duane (2011) | Not reported | Not reported | Not reported | Not reported |
| Duane (2013) | Not reported | None | Not reported | Not reported |
| Ehrlich (2009) | 16 | 16 | 0 | Complete – case analysis |
| Engelbart (2021) | Not reported | Not reported | Not reported | Not reported |
| Enthoven (2016) | Not clearly reported | Not clearly reported (missing values ranged from 0% to 13%) | 6 | Complete – case analysis |
| Ghelichkhani (2021) | Not reported | Not reported | Not reported | Complete – case analysis |
| Henschke (2009) | 21 at the 6 weeks follow-up, 17 at the 3 months follow-up, and 5 at the 12 months follow-up | 0 | 21 at the 6 weeks follow-up, 17 at the 3 months follow-up, and 5 at the 12 months follow-up | Complete – case analysis |
| Hercz (2019) | None | None | None | Not applicable |
| Ikemoto (2022) | None | None | None | Not applicable |
| Inaba (2015) | Not reported | Not reported | Not reported | Complete – case analysis |
| Inagaki (2018) | 265 | Not reported | 265 | Complete – case analysis |
| Khera (2022) | 264 patients | Not reported | Not reported | Primary analysis using a complete case – approach. Sensitivity analysis using 10 multiple imputed datasets |
| Leonard (2011) | Cases: 394  Random controls: 158  Mechanism of injury controls: 603  Emergency medical services control: 110 | Not reported | Not reported | Complete – case analysis  Sensitivity analysis performed using multiple imputation |
| Roux (2007) | 27 patients | 13 patients | 14 patients | Complete – case analysis |
| Singh (2011) | 10 controls | Not reported | Not reported | Complete – case analysis |
| Stiell (2001) | 577 | Not reported | 577 | Complete – case analysis |
| Stiell (2003) | 1480 | 845 (missing range of motion of the rule) | 635 | Primary analysis as a complete – case analysis. Secondary analysis considers the patients with missing predictors as all tested positive or all tested negative to the rule |
| Stiell (2010) | 181 | 181 (missing range of motion of the rule) | Not reported | Complete – case analysis |
| Vaillancourt (2009) | 446 for paramedics  764 for study investigators | 2 for paramedics  320 for study investigators | 444 for paramedics  444 for study investigators | Complete – case analysis |
| Vaillancourt (2023) | 13 for paramedics  192 for study investigators | 179 for study investigators | 13 for paramedics  13 for study investigators | Complete – case analysis |
