# Supplementary material for: Diagnostic prediction models for spinal fractures in individuals with spinal pain or trauma: a systematic review and meta-analysis
Source: eClinicalMedicine. 2025 Aug 26;88:103456. doi: 10.1016/j.eclinm.2025.103456 (PMC12572814; doi:10.1016/j.eclinm.2025.103456)
Supplement: Supplementary Material 10 [file mmc10.docx]

| **First author (year)** | **Number patients/events** | **FACTORS THAT MAY DECREASE THE QUALITY** | | | | | | **FACTORS THAT MAY INCREASE THE QUALITY** | | **RESULTS** | **Certainty of the evidence** |
| --- | --- | --- | --- | --- | --- | --- | --- | --- | --- | --- | --- |
|  |  | **Phase of investigation** | **Study limitations** | **Inconsistency** | **Indirectness** | **Imprecision** | **Publication bias** | **Moderate or large effect size** | **Exposure-response gradient** |  |  |
| Athinartrattanapong (2021) | 375/29 | Development | Very serious | / | No | Yes | No | No | No | Low risk (score of 0): **LR+** 0.18 (95% CI 0.05 to 0.56)  Moderate risk (score between 1 and 5): **LR+** 1.46 (95% CI 1.09 to 1.96)  High risk (score between 6 and 11): **LR+** 7.16 (95% CI 2.82 to 18.19) | **VERY LOW** |
| Athinartrattanapong (2021) | 375/29 | Development | Very serious | / | No | Yes | No | Yes | No | **AUC** 0.83 (95% CI 0.74 to 0.91) | **VERY LOW** |
| Bub (2005) | 210/103 | Development | Very serious | / | No | / | No | Yes | No | **AUC** 0.82 | **LOW** |
| Clark (2016) | 197/64 | Development | Very serious | / | Serious | Yes | No | Yes | No | **AUC** 0.85 (95% CI 0.79 to 0.92) | **VERY LOW** |
| Clark (2016) | 197/64 | Development | Very serious | / | Serious | Yes | No | No | No | With a cut-off of 0.39:  - **Sensitivity** 77%  - **Specificity** 78% | **VERY LOW** |
| Cook (2013) | 162/11 | Development | Very serious | / | No | No | No | Yes | No | Presence of 1 of 6 predictors:  - **Sensitivity** 100% (95% CI 100% to 100%)  - **Specificity** 15% (95% CI 9% to 21%)  Presence of 2 of 6 predictors:  - **Sensitivity** 100% (95% CI 100% to 100%)  - **Specificity** 50% (95% CI 42% to 58%)  Presence of 3 of 6 predictors:  - **Sensitivity** 91% (95% CI 74% to 100%)  - **Specificity** 84% (95% CI 78% to 90%)  Presence of 4 of 6 predictors:  - **Sensitivity** 64% (95% CI 35% to 92%)  - **Specificity** 98% (95% CI 96% to 100%)  Presence of 5 of 6 predictors:  - **Sensitivity** 18% (95% CI 0% to 41%)  - **Specificity** 100% (95% CI 100% to 100%) | **LOW** |
| Duane (2011) (Developed model) | 3201/192 | Development | Very serious | / | No | / | No | Yes | No | **AUC** 0.77 | **LOW** |
| Duane (2013) (Developed model) | 5182/324 | Development | Very serious | / | No | / | No | Yes | No | **AUC** 0.75 | **LOW** |
| Duane (2013) (Developed model) | 5182/324 | Development | Very serious | / | No | / | No | Yes, for sensitivity | No | **Sensitivity** 99.07%  **Specificity** 11.57% | **LOW** |
| Ehrlich (2009) | 125/7 | External validation | Very serious | / | Serious | / | No | Yes | No | **Sensitivity** 86%  **Specificity** 94% | **LOW** |
| Engelbart (2021) (Model 1) | 1605/178 | Development | Very serious | / | Serious | / | No | Yes | No | **AUC** 0.83 | **VERY LOW** |
| Engelbart (2021) (Model 1) | 1605/178 | Development | Very serious | / | Serious | No | No | Yes, for specificity | No | **Sensitivity** 61.1% (95% CI 53.2% to 68.9%)  **Specificity** 93.7% (95% CI 92.3% to 95.0%) | **VERY LOW** |
| Engelbart (2021) (Model 2) | 1605/178 | Development | Very serious | / | Serious | / | No | No | No | **AUC** 0.66 | **VERY LOW** |
| Engelbart (2021) (Model 2) | 1605/178 | Development | Very serious | / | Serious | No | No | Yes, for specificity | No | **Sensitivity** 8.1% (95% CI 2.4% to 13.9%)  **Specificity** 97.4% (95% CI 96.5% to 98.2%) | **VERY LOW** |
| Enthoven (2016) | 669/33 | Development | Very serious | / | Serious | No | No | Yes | No | **AUC** 0.78 (range 0.69 to 0.87) | **VERY LOW** |
| Enthoven (2016) | 669/33 | Development | Very serious | / | Serious | No | No | Yes | No | ≥ 1 positive feature:  - **Sensitivity** 88% (95% CI 77% to 995)  - **Specificity** 42% (95% CI 38% to 46%)  ≥ 2 positive features:  - **Sensitivity** 70% (95% CI 54% to 85%)  - **Specificity** 81% (95% CI 78% to 84%)  ≥ 3 positive features:  - **Sensitivity** 30% (95% CI 15% to 46%)  - **Specificity** 95% (95% CI 93% to 97%) | **VERY LOW** |
| Henschke (2009) | 1172/8 | Development | Very serious | / | Serious | Yes | No | Yes | No | **AUC** 0.83 (95% CI: 0.65 to 1) | **VERY LOW** |
| Henschke (2009) | 1172/8 | Development | Very serious | / | Serious | / | No | Yes | No | One positive feature:  - **Sensitivity** 88%  - **Specificity** 50%  Two positive features:  - **Sensitivity** 63%  - **Specificity** 96%  Three positive features:  - **Sensitivity** 38%  - **Specificity** 100% | **VERY LOW** |
| Hercz (2019) | 1049/36 | Development | Very serious | / | Serious | No | No | Yes | No | **Sensitivity** 100% (95% CI 91.2% to 100%) **Specificity**: 53.1% (95% CI 50.5% to 55.7%) | **VERY LOW** |
| Ikemoto (2022) | 80/40 | Development | Very serious | / | No | / | No | Yes | No | **AUC** 0.88 | **LOW** |
| Ikemoto (2022) | 80/40 | Development | Very serious | / | No | / | No | Yes | No | Sum score of 2:  - **Sensitivity** 97.5%  - **Specificity** 32.5%  Sum score of 3:  - **Sensitivity** 87.5%  - **Specificity** 75.0%  Sum score of 4:  - **Sensitivity** 65.0%  - **Specificity** 95.0% | **LOW** |
| Inaba (2015) (Model 1) | 3065/264 | Development | Very serious | / | Serious | No | No | Yes | No | **AUC** 0.81 (95% CI 0.78 to 0.83) | **VERY LOW** |
| Inaba (2015) (Model 2: rule) | 3065/264 | Development | Very serious | / | Serious | / | No | Yes, for sensitivity | No | **Sensitivity** 98.9%  **Specificity** 29.0% | **VERY LOW** |
| Inagaki (2018) | 927/38 | Development | Very serious | / | Serious | No | No | Yes, for sensitivity | No | **Sensitivity** 100% (95% CI 90.8% to 100%)  **Specificity** 51.9 % (95% CI 48.6% to 55.2%) | **VERY LOW** |
| Inagaki (2018) | 927/38 | External validation | Very serious | / | Serious | Yes | No | Yes, for sensitivity | No | **Sensitivity** 92.1% (95% CI 79.2% to 97.3%)  **Specificity** 58.6 % (95% CI 55.4% to 61.9%) | **VERY LOW** |
| Khera (2022) | 1601/202 | Development | Very serious | / | No | / | No | Yes | No | **AUC** 0.78 | **LOW** |
| Khera (2022) | 1601/202 | Development | Very serious | / | No | / | No | No | No | Sensitivity 72.4%  **Specificity** 72.9% | **VERY LOW** |
| Khera (2022) | 1601/202 | Development | Very serious | / | No | / | No | Yes | No | **Calibration slope** 1.0 | **LOW** |
| Leonard (2011) (Model 1: Mechanism of injury controls) | 1012/540 | Development | Very serious | / | Serious | No | No | Yes | No | **Sensitivity** 94% (95% CI 91% to 96%)  **Specificity** 32% (95% CI 29% to 35%) | **VERY LOW** |
| Leonard (2011) (Model 2: Emergency medical services controls) | 702/540 | Development | Very serious | / | Serious | No | No | Yes | No | **Sensitivity** 92% (95% CI 89% to 94%)  **Specificity** 35% (95% CI 32% to 38%) | **VERY LOW** |
| Roux (2007) | 397/not clear | Development | Very serious | / | Serious | / | No | Yes | No | **AUC** 0.77 | **VERY LOW** |
| Singh (2011) | 773/261 | Development | Very serious | / | No | / | No | Yes | No | **AUC** 0.88 | **LOW** |
| Singh (2011) | 773/261 | Development | Very serious | / | No | / | No | Yes | No | **Sensitivity** 64%  **Specificity** 93% | **LOW** |
| Stiell (2001) (Model 1: logistic regression model) | 8924/515 | Development | Very serious | / | Serious | / | No | Yes | No | **AUC** 0.91 | **VERY LOW** |
| Stiell (2001) (Model 2: Canadian C-spine rule) | 8924/515 | Development | Very serious | / | Serious | No | No | Yes | No | **Sensitivity** 100% (95% CI 98% to 100%)  **Specificity** 42.5% (95% CI 40% to 44%) | **VERY LOW** |
| Stiell (2010) | 3411/41 | External validation | Very serious | / | Serious | Yes | No | Yes, for sensitivity | No | **Sensitivity** 90.2% (95% CI 76% to 95%)  **Specificity** 43.9% (95% CI 42% to 46%) | **VERY LOW** |
| Vaillancourt (2009) | 1947/12 | External validation | Very serious | / | Serious | Yes | No | Yes, for sensitivity | No | **Sensitivity** 100% (95% CI 74% to 100%)  **Specificity** 37.7% (95% CI 36% to 40%) | **VERY LOW** |
| Vaillancourt (2010) | 4021/11 | External validation | Very serious | / | Serious | Yes | No | Yes, for sensitivity | No | **Sensitivity** 90.9% (95% CI 58.7% to 99.8%)  **Specificity** 66.5% (95% CI 65.1% to 68.0%) | **VERY LOW** |
